# Testing, Experimental Design, and Numerical Analysis of Nanomechanical Properties in Epoxy Hybrid Systems Reinforced with Carbon Nanotubes and Graphene Nanoparticles

**DOI:** 10.3390/polym16233420

**Published:** 2024-12-05

**Authors:** Giovanni Spinelli, Rosella Guarini, Todor Batakliev, Liberata Guadagno, Marialuigia Raimondo

**Affiliations:** 1 Faculty of Transport Sciences and Technologies, University of Study “Giustino Fortunato”, Via Raffaele Delcogliano 12, 82100 Benevento, Italy; 2 Open Laboratory on Experimental Micro and Nano Mechanics, Institute of Mechanics, Bulgarian Academy of Sciences, Acad. G. Bonchev Str., Block 4, 1113 Sofia, Bulgaria; rgrosagi@gmail.com (R.G.); todorbat@gmail.com (T.B.); 3 Department of Industrial Engineering, University of Salerno, Via Giovanni Paolo II, 84084 Fisciano, Italy; lguadagno@unisa.it

**Keywords:** nanoindentation, epoxy resin, mechanical properties, hybrid systems

## Abstract

Hybrid nanocomposites incorporating multiple fillers are gaining significant attention due to their ability to enhance material performance, offering superior properties compared to traditional monophase systems. This study investigates hybrid epoxy-based nanocomposites reinforced with multi-walled carbon nanotubes (MWCNTs) and graphene nanosheets (GNs), introduced at two different weight concentrations of the mixed filler, i.e., 0.1 wt% and 0.5 wt% which are, respectively, below and above the Electrical Percolation Threshold (EPT) for the two binary polymer composites that solely include one of the two nanofillers, with varying MWCNTs:GNs ratios. Mechanical properties, such as contact depth, hardness, and reduced modulus, were experimentally assessed via nanoindentation, while morphological analysis supported the mechanical results. A Design of Experiments (DoE) approach was utilized to evaluate the influence of filler concentrations on the composite’s mechanical performance, and Response Surface Methodology (RSM) was applied to derive a mathematical model correlating the filler ratios with key mechanical properties. The best and worst-performing formulations, based on hardness and contact depth results, were further investigated through detailed numerical simulations using a multiphysics software. After validation considering experimental data, the simulations provided additional insights into the mechanical behavior of the hybrid composites. This work aims to contribute to the knowledge base on hybrid composites and promote the use of computational modeling techniques for optimizing the design and mechanical performance of advanced materials.

## 1. Introduction 

Materials are central to the design and function of nearly every product we use, making it essential to carefully choose the right material for each application. In industries such as aerospace, automotive, and construction, material selection plays a crucial role [1]. While metals, alloys, and ceramics are commonly used in manufacturing, their high density can limit their effectiveness in certain applications. For instance, the aforementioned fields require lightweight materials to improve efficiency and reduce costs [2]. The limitations of traditional materials can be addressed by using composite materials, which provide comparable properties to metals and alloys. Composites are particularly valued for their lightweight nature, affordability, and exceptional strength-to-weight ratio [3]. 

Thermosetting polymers are favored in both academia and industry for their superior stiffness and strength compared to thermoplastics [4]. Epoxy is the most frequently used thermosetting matrix. Its distinctive properties, including low residual stress due to minimal shrinkage during curing and the low pressure needed for production, set it apart from other thermosetting resins [5,6]. Epoxy finds broad engineering applications in composites, surface coatings, molding processes, construction materials, and numerous other areas.

Nanostructured materials with varying degrees of dimensionality have garnered significant attention in recent composite research. In particular, carbon-based nanomaterials, including carbon nanotubes (CNTs) and graphene nanoplatelets (GNPs), are known for their exceptional thermo-mechanical and electrical characteristics [7,8]. Their high aspect ratio further enhances their suitability as fillers in polymer nanocomposites, making them ideal for use in advanced material applications [9].

The success of these improvements relies not only on the structure, size, porosity, and interface of the carbon nanostructures within the epoxy matrix [10] but also on functionalization techniques that can be employed to increase their compatibility and dispersion in the matrix [11]. In recent times, a new trend in composite materials has begun to emerge, specifically multi-phase composites: carbon nanotubes and graphene nanoplatelets are integrated into conventional composites to provide reinforcement at multiple scales [12]. 

Different literature studies proposed that graphene oxide (GO) can be utilized to enhance the dispersion of carbon nanotubes (CNTs) within the polymer matrix [13,14]. Similarly, some researchers [15,16] showed that graphene-based materials, such as graphene oxide or graphene nanoplatelets, can significantly improve the percolation threshold in nanocomposites. The addition of graphene sheets helps create a more interconnected conductive network, which lowers the percolation threshold compared to composites using CNTs alone. This improved network formation is due to graphene’s high aspect ratio and large surface area, which facilitate better filler–filler interactions and charge transport, enhancing the composite’s electrical conductivity.

Maiti et al. demonstrated a novel technique that involves in situ polymerization of styrene/multi-walled carbon nanotubes (MWCNTs) in the presence of suspension polymerized polystyrene (PS)/graphite nanoplate (GNP) microbeads for the preparation of electrically conducting PS/MWCNT/GNP nanocomposites with very high (~20.2 dB) EMI shielding value at extremely low loading of MWCNTs (~2 wt%) and GNP (~1.5 wt%) [17]. More in general, this incorporation of both carbon-based filler (CNTs and GNPs) reveals the synergistic effects derived from strong π–π interactions and enhanced interfacial bonding with the polymer matrix [18,19], which, in turn, lead to a remarkable improvement in the overall properties of the corresponding composites. More specifically, the approach of adding hybrid carbon nanofillers with varying geometries has proven to be an efficient method for enhancing the mechanical properties of composite materials, such as tensile strength, modulus of elasticity, creep, and thermal stability [20,21]. Yu et al., in their study [22], proved that a hybrid nanoscale filler made of single-walled carbon nanotubes and graphite nanoplatelets delivers a synergistic improvement in the thermal conductivity of epoxy composites. This enhancement is attributed to the creation of a more effective percolating network, which significantly lowers thermal interface resistance. Research efforts are often focused on the development of hybrid nanocomposites incorporating a combination of carbon nanotubes (CNTs) and a second carbon-based filler to improve a selected property, such as electrical conductivity, and maintain balanced mechanical properties, all while reducing the overall production cost. Epoxy-based nanocomposites with varying amounts of CNTs and carbon black (CB) as conductive fillers were developed and tested for electrical and mechanical properties [23]. The inclusion of CNTs improved electrical conductivity, achieving a low percolation threshold at 0.2 wt% CNTs and CB. Additionally, CB enhanced ductility and fracture toughness, showcasing a synergistic effect from the combination of these two fillers with distinct geometries and dispersion characteristics. Prasad et al. highlighted the remarkable synergy when two different nanocarbons, such as nanodiamond and graphene, are combined in a polymer matrix based on polyvinyl alcohol [24]. The resulting composites show a dramatic increase (evaluated by the innovative nanoindentation technique) in stiffness and hardness, up to 400%, compared to those with only one nanocarbon reinforcement. Remarkable impacts of graphene nanoplatelets and carbon nanotubes on the mechanical (tensile strength) and thermal characteristics of epoxy-based composites were also observed by Yang et al. [25]. In Araby et al., the combination of MWCNTs and GNPs in ethylene–propylene–diene rubber resulted in a significant synergy, improving tensile strength, Young’s modulus, and tear strength by up to 825% compared to lower increases when only GNPs were used [26]. This demonstrates the complementary effect of the two carbon fillers. By adjusting the mass ratio of carbon nanotube to graphene within polymer matrices, one can effectively regulate their electrical and thermal conductivity, percolation threshold, and mechanical and other key properties. A synergistic enhancement in the flexural properties of epoxy composites was observed when carbon nanotubes (CNTs) and graphene nanoplatelets (GNPs) were combined in an 8:2 ratio and dispersed by ultrasonication [27]. Min et al. observed unique synergistic effects of graphene oxide and carbon nanotube hybrids on the tribological properties of polyimide nanocomposites, which come to the best as the ratio of GO and CNTs is 3:1 [28]. In our earlier research, we examined epoxy-based hybrid systems by integrating multi-walled carbon nanotubes (MWCNTs) and graphene nanosheets (GNs) at two set mixed filler concentrations: one below (0.1 wt%) and the other above (0.5 wt%), both at the same different MWCNTs:GNs combination ratios [29]. The aim of this investigation was to explore the relationship between the synergistic properties, assessed through thermogravimetric and dynamic mechanical analyses, in the designed epoxy systems containing MWCNTs and GNs and the dispersion state of the fillers across various length scales. This current research not only revisits these previous formulations but also expands upon them by providing an in-depth nanomechanical characterization of various composite samples. The experimental work was conducted by using the innovative nanoindentation technique, allowing for precise measurements of key mechanical properties such as hardness, reduced modulus, and contact depth. A key focus of the study is the investigation of the influence of different MWCNTs:GNs ratios on these mechanical properties, using, for the first time in the literature to the best of our knowledge, a statistical approach based on the Design of Experiments (DoE) methodology. Moreover, the response surface method provided valuable insights by establishing a numerical relationship between the nanoindentation-derived mechanical properties and the filler weight ratios. This statistical approach not only enhances our understanding of how these nanocomposites behave at different filler ratios but also offers crucial design guidance for improving the development of new materials. It allows researchers and engineers to predict performance trends, optimizing material compositions for specific applications. In addition to the experimental work, the study integrates finite element method (FEM) simulations with the nanoindentation data, providing a dual approach that reinforces the accuracy and reliability of the findings. By closely mirroring the experimental conditions in the simulations, the study minimizes potential errors, ensuring an excellent correlation between simulated and experimental results. This successful validation of the model underscores the power of simulations to predict nanomechanical properties before physical testing, streamlining the design process and offering a more efficient path to material development with enhanced performance.

The uniqueness of this study lies in its comprehensive approach to investigating hybrid nanocomposites reinforced with MWCNTs and GNs. By systematically varying the filler concentrations and MWCNTs:GNs mix ratios and also examining their influence on mechanical properties through experimental nanoindentation, this work offers new insights into the behavior of hybrid composites. Additionally, the integration of a Design of Experiments (DoE) approach and Response Surface Methodology (RSM) to develop a predictive model provides a robust framework for understanding the relationship between filler ratios and mechanical performance. The use of multiphysics simulations further complements the experimental findings, offering a detailed, quantitative perspective on the mechanical behavior of the composites. This novel approach not only advances the understanding of hybrid nanocomposites but also demonstrates the potential of computational modeling for optimizing the design and performance of advanced materials in a wide range of applications.

## 2. Materials

### 2.1. Materials

The epoxy matrix, referred to as EP, consists of a mixture of tetraglycidyl methylene dianiline (TGMDA) and 1,4-butanedioldiglycidylether (BDE) in a weight concentration of 80 wt% and 20 wt%, respectively, in which the stoichiometric amount of the curing agent 4,4′-diaminodiphenyl sulfone (DDS) is solubilized. All chemicals were procured from Sigma-Aldrich (Milan, Italy). In this research, multi-walled carbon nanotubes (MWCNTs), purchased from Nanocyl S.A. (Sambreville, Belgium) and graphene nanosheets (GNs) (Asbury graphite grade 3759, Asbury Carbons, Asbury, NJ, USA)) were employed as conductive carbon-based nanofillers.

**Figure 1:**
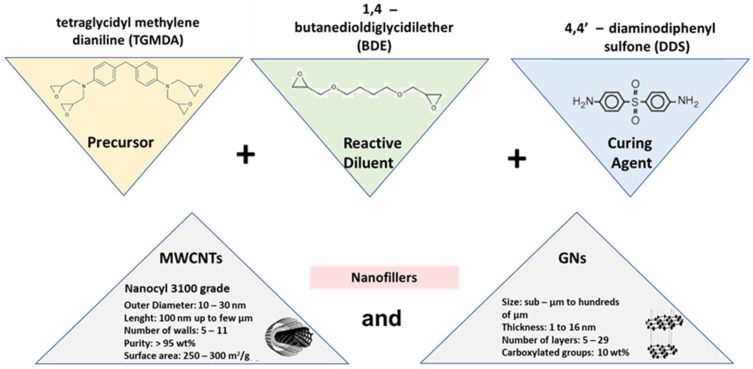
Chemical particulars of the precursor, reactive diluent, and curing agent, as well as main features of both carbon-based nanofillers: MWCNTs and GNs.

Figure 1 reports the molecular configurations of the epoxy precursor (TGMDA), reactive diluent (BDE), and curing agent (DDS), as well as the main characteristics of both fillers.

The hybrid nanocomposites were formulated by uniformly incorporating and dispersing both one-dimensional (1D) and two-dimensional (2D) nanofillers into the epoxy matrix (TGMDA + BDE) at 90 °C for 20 min using a Hielscher UP200S (200 W, 24 kHz) ultrasonic system (Hielscher Ultrasonics GmbH, Teltow, Germany). Afterward, the temperature was raised to 120 °C, and the curing agent DDS was added to the mixture, which was continuously stirred magnetically until DDS was completely dissolved. The resulting liquid nanocomposite formulations were then degassed at around 100 °C under vacuum for 1 h to eliminate air bubbles trapped within the mixture. Solidification of the unfilled EP resin and all hybrid epoxy samples was carried out in two isothermal steps: the first stage involved heating at 125 °C for 1 h, followed by a second stage at elevated temperatures reaching 200 °C for 3 h. This process uses lower temperatures and shorter times in the initial curing stage, followed by higher temperatures for the final stage, reflecting standard industrial conditions. 

In this study, the hybrid epoxy samples were designated with the acronym HYB X% (MWCNTs:GNs). More in detail, X% denotes the overall weight percentages of the nanofiller mix (0.1 and 0.5 wt%, which are, respectively, below and above the EPT for the two binary single-filler epoxy systems. The labeling (MWCNTs:GNs) specifies one of the five weight mix ratios (1:1, 1:2, 1:5, 2:1, 5:1) between the two fillers.

Table 1 provides the corresponding percentage by weight of each nanofiller present in the mix of the two nanofillers, MWCNTs and GNs, for each combination ratio.

### 2.2. Density Measurement

The hydrostatic balance KERN_AEJ_ -YDB-03 (KERN & SOHN GmbH, Balingen, Germany), based on the Archimedes principle, was used for measuring the density of all produced hybrid nanocomposites by measuring their weight in air and when submerged in a liquid. In our experimental measurements, ethanol (C_2_H_5_OH 96%—M:46.07 g/mol) was adopted as a liquid. Its temperature at the time of measurement was 20 °C.

### 2.3. Nanoindentation Tests

**Figure 2:**
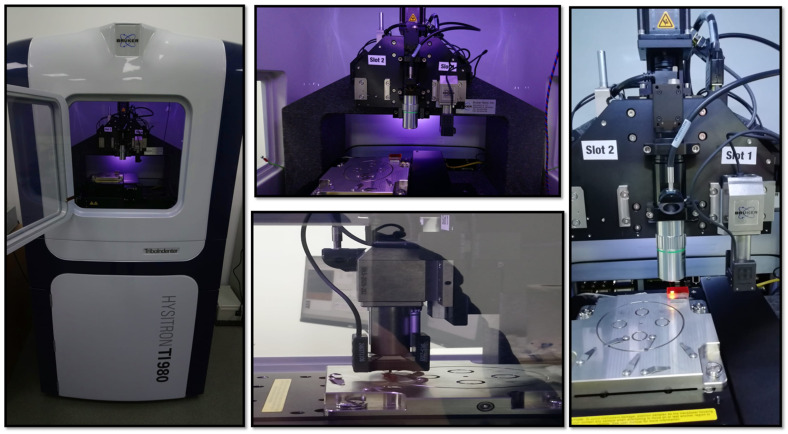
Nanomechanical test system for innovative material characterization.

Nanoindentation tests were performed to assess the mechanical properties using a Hysitron TI 980 instrument (Bruker, Billerica, MA, USA), as shown in Figure 2, which showcases high-quality images of the measurement instrument successfully adopted in one of our previous works [30]. The setup included a 2D transducer assembly (measuring both normal and lateral forces) with a Berkovich probe with a radius of curvature of approximately 150 nm.

**Figure 3:**
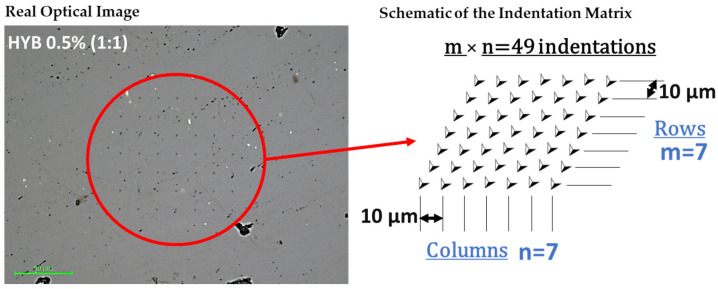
Real optical image of nanoindentation test trace performed on the surface of HYB 0.5% (1:1) sample and schematic representation of the indentation matrix.

The tip area was calibrated using a reference specimen of fused quartz with a known elastic modulus of 69.6 GPa. Nanoindentation tests were conducted in load-controlled mode, applying a peak load of 10,000 μN. Due to the viscoelastic behavior of the samples, a holding segment was added to the load profile. Each test involved 49 indentations arranged in a 7 × 7 grid with 10 μm spacing between indents to collect statistical data and assess the variability in load–displacement curves caused by surface roughness (see Figure 3).

**Figure 4:**
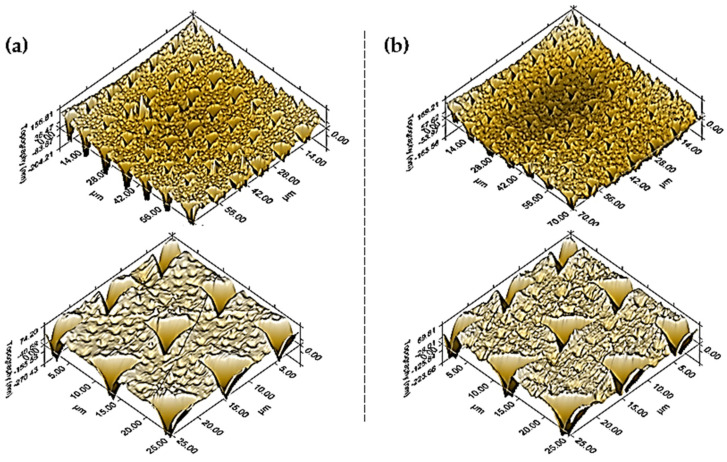
Three-dimensional SPM images of the nanoindentation test traces captured on the surfaces of the samples: (**a**) HYB 0.5% (1:1) and (**b**) HYB 0.5% (5:1).

The in situ high-resolution scanning probe microscopy (SPM) technique, integrated with the nanomechanical test apparatus, was used to capture topographical images of the nanoindentation test traces on the two selected samples from this study, HYB 0.5% (1:1) and HYB 0.5% (5:1), as illustrated in Figure 4a and b, respectively. 

### 2.4. Multiphisic Simulation Study of Naomechanical Properties

This study also investigated the nanomechanical behavior, including contact depth and local displacement, hardness, and Von Mises stress, of some selected composites using the 3D finite element method (FEM) in the COMSOL Multiphysics^®^ (version 6.1) environment. A Berkovich tip, modeled in 3D CAD software (FUSION 360, version 2024) based on real manufacturer data, was used to simulate experimental nanoindentation, thus addressing, as already highlighted in our previous work [30], the limitations of simpler tip models often used in the literature studies [31,32,33,34,35]. 

**Figure 5:**
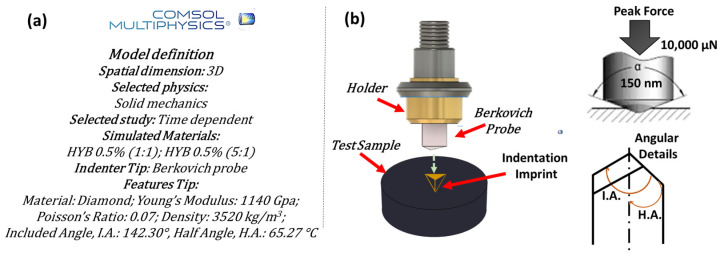
(**a**) Key model definitions for the numerical analysis. (**b**) Schematic illustration of the case study numerically investigated.

For reasons of clarity and conceptual simplicity, Figure 5a outlines the key model definitions chosen for the simulations, while Figure 5b presents a schematic illustration of the case study under consideration. 

The type of tip and its geometric characteristics, as previously described in our earlier work [30], which the authors recommend for further details, are crucial in determining the mechanical properties of the materials it contacts and indents. Compared to the corresponding figure in our previous work [30], this new figure emphasizes angular details more clearly, such as the included angle and the half-angle, which are particularly relevant to the specific type of tip used in the simulation.

**Figure 6:**
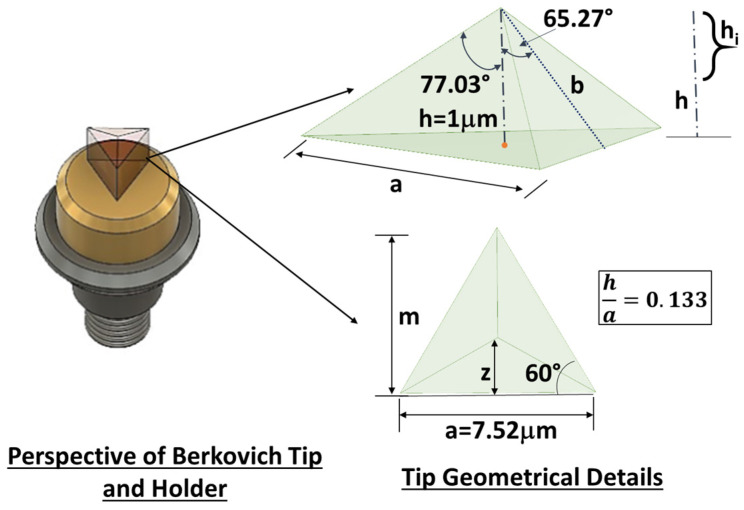
Geometrical features of the Berkovich’s indenter tip.

Figure 6 highlights the key features that enable the determination of the projected area (A_proj_) using straightforward geometric considerations. As in our previous work [30], this figure emphasizes critical details, ensuring a clear and concise representation of the adopted tip while enhancing the understanding of the underlying geometric principles already described in greater detail in [30].

(1) $$A_{proj}=3\sqrt{3}h_{i}^{2}{tan}^{2}65.27{}^{\circ}≅24.56\cdot h_{i}^{2}$$

which, in turn, is used to calculate the material’s hardness (H) according to the Doerner–Nix model mathematically expressed by the following relationship [36]:

(2) $$H=\frac{L_{max}}{A_{proj}}$$

In words, the formula of the hardness H translates to the ratio between the maximum applied load (L_max_) divided by the projected contact surface.

### 2.5. Design of Experiments (DoE)

**Figure 7:**
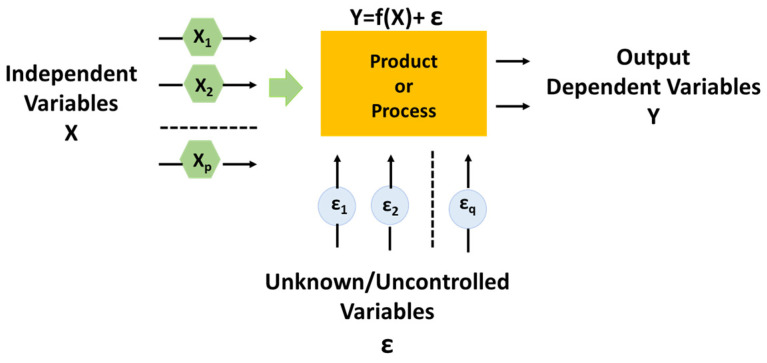
Schematic view of the experiment design.

Experiment design treats (see Figure 7) a process as a “black box” with inputs (independent variables) and outputs (dependent variables, Y). By carefully controlling the inputs and observing the changes in outputs, the goal is to isolate the effects of these controlled variables, minimizing the impact of unknown or uncontrolled factors [37,38]. This approach allows for clear conclusions about how the controlled inputs influence the outputs. Therefore, the best parameter settings can be adopted to improve the desired output.

In the present study, the Design of Experiments (DoE) is employed to evaluate the impact of two nanofiller amounts (i.e., wt% _MWCNTs_ and wt% _GNs_) in their weight ratio MWCNTs:GNs on certain mechanical properties investigated experimentally, namely the contact depth, hardness, and the reduced modulus.

For a successful application of DoE, it is essential to establish an appropriate discretization level for the input variables. In our study, the input variable vector $\bar{x}$ has been defined as follows:

(3) $$\bar{x}=({wt\%}_{MWCNTs}:x,x:{wt\%}_{GNs})\epsilon R^{2}$$

where a discretization on 3 levels is applied to the selected input factors within the following values: x = 1 wt%, x = 2 wt%, and x = 5 wt% for both MWCNTs and GNs, respectively. 

More in details, the considered values are as follows:

(4) $${{wt\%}_{MWCNTs}}_{1}:x=1:x,{{wt\%}_{MWCNTs}}_{2}:x=2:x,{{wt\%}_{MWCNT}}_{s3}:x=5:x$$

(5) $$x:{{wt\%}_{GNs}}_{1}=x:1,x:{{wt\%}_{GNs}}_{1}=x:2,x:{{wt\%}_{GNs}}_{1}=x:5$$

As a result, the compact set D representing the variable space is mathematically defined as follows:

(6) $$D={wt\%}_{MWCNT}:x\times x:{wt\%}_{GN}\subset R^{2}$$

while the dependent variable (i.e., the nanomechanical property) is assessed for each ordered pair $\left({wt\%}_{MWCNTs}:x,x:{wt\%}_{GNs}\right)$ of the input variable vector, i.e.,

(7) $$\left({wt\%}_{MWCNTs}:x,x:{wt\%}_{GNs}\right)\;\epsilon \;D.$$

### 2.6. Response Surface Methodology (RSM)

Response Surface Methodology (RSM), originally developed by Box and Wilson in the early 1950s, remains a highly utilized mathematical technique grounded in the principles of Design of Experiments (DoE) [39]. This method is applied to forecast the relationship between several design variables and the corresponding experimental results. In cases where the precise structure of the performance function (P.F.) is not known, RSM seeks to estimate the response surface (R.S.), aiming to pinpoint regions of optimal response as design inputs are modified. The response surface is commonly represented in the following form:

(8) $$R.S.=f\;\left(X_{1},{\;X}_{2},\;\ldots X_{n}\right)+\varepsilon$$

In this context, *f* represents the mathematical relationship between the response surface (R.S.) and the independent input variables (*Xi*), while ε accounts for the experimental error, which is assumed to follow a normal distribution with a mean of zero and a constant variance. Polynomial models are frequently applied to predict surface behavior; either first-order (linear) or second-order (quadratic) models, as utilized in this study, are generally sufficient for analyzing performance across a range of problems. This approach is particularly effective when the outcome depends on two or three input variables, such as, in our case, the amount of one-dimensional filler and two-dimensional filler in their weight ratio MWCNTs:GNs [40,41].

In mathematical terms, the quadratic polynomial model (*n* = 2) can be expressed by the following equation:

(9) $$R.S.=\beta_{0}+\sum_{i=1}^{n}\beta_{i}x_{i}+\sum_{i=1}^{n}\beta_{ii}x_{i}^{2}+\sum_{i=1}^{n-1}\sum_{j=i+1}^{n}\beta_{ij}x_{i}x_{j}$$

Here, *x_i_*, *x_j_* denote the independent input parameters, *β_0_* represents the intercept coefficient, while *β_i_*, *β_ii_*, and *β_ij_* denote the coefficients for linear, quadratic, and interaction terms, respectively. These coefficients are determined using the least squares method.

In the present study, the functions of interest for the Response Surface Methodology (RSM) are represented by the nanomechanical properties experimentally investigated: reduced modulus (Er), hardness (H), and contact depth (CD). For the sake of generalization, we introduce the notation “nanomechanical property” (NP), which applies to all three cases. The independent variables are represented by the weight percentages of multi-walled carbon nanotubes (MWCNTs) and graphene nanosheets (GNs), specifically referring to their weight ratio MWCNTs:GNs. Therefore, the goal of RSM is to find a reliable analytical relationship between the specified nanomechanical properties (NP) and the identified independent variables: $NP=f\left(x:GNs,MWCNTs:x\right)=f\left(x_{1},x_{2}\right)$ for a more concise and suitable mathematical representation. Based on Equation (7), the quadratic polynomial that approximates the function of interest *NP* is given by the following:

(10) $$NP=f\left(x_{1},x_{2,}\right)=\beta_{0}+\beta_{1}x_{1}+\beta_{2}x_{2}+\beta_{12}x_{1}x_{2}+\beta_{11}x_{1}^{2}+\beta_{22}x_{2}^{2}$$

### 2.7. Morphological Analysis

The morphological characterization of the nanocomposites HYB 0.5% (1:1) and HYB 0.5% (5:1) was carried out by Field Emission Scanning Electron Microscopy (FESEM) and Tunneling Atomic Force Microscopy (TUNA) technique. The sample slices obtained from solid samples by a sledge microtome were etched before the observation by FESEM and TUNA to discover the carbon nanofillers because they are deprived of the surrounding resin layer, which is consumed by the oxidizing solution. The etching reagent was prepared by stirring 1.0 g potassium permanganate in a solution mixture of 95 mL sulfuric acid (95–97%) and 48 mL orthophosphoric acid (85%). The filled resins were immersed into the fresh etching reagent at room temperature and held under agitation for 36 h. Subsequent washings were performed using a cold mixture of two parts by volume of concentrated sulfuric acid and seven parts of water. Afterward, the samples were washed again with 30% aqueous hydrogen peroxide to remove any manganese dioxide. The samples were finally washed with distilled water and kept under vacuum for 5 days before being subjected to morphological analysis. For FESEM analysis, we used a LEO 1525 model (Carl Zeiss SMT AG, Oberkochen, Germany). All the samples were placed on a carbon tab previously stuck to an aluminum stub (Agar Scientific, Stansted, UK) and were covered with a 250 Å thick gold film using a sputter coater (Agar mod. 108 A). Information on topography and local electrical current of the hybrid nanocomposites was obtained by the TUNA technique operating in contact mode and using platinum-coated probes with nominal spring constants of 35 N m^−1^ and an electrically conductive tip of 20 nm. The TUNA module measures ultra-low currents (<1 pA) ranging from 80 fA to 120 pA circulating through the conductive tip to the investigated samples kept at a fixed DC bias. In this work, we used a DC sample bias from 1 V to 2 V, a current sensitivity of 1 pA/V, a scan rate of 0.500 Hz s^−1^, and the number of pixels in X and Y (samples/line) set to 512. In this work, the electrical characterization at the nanoscale level was carried out without grounding the samples. The TUNA images were analyzed using the Bruker software Nanoscope Analysis 1.80 (Build R1.126200). 

## 3. Results and Discussion

The mechanical properties of materials are crucial considerations in the design of structural elements. Nanoindentation is a widely employed method for evaluating these characteristics at the nanoscale. This technique involves monitoring the displacement of a precisely calibrated indenter as it penetrates the surface of a sample under controlled forces. The resulting force–displacement curve provides insights into material properties, such as elastic modulus and hardness, through approaches like the Oliver and Pharr method [42]. Moreover, several other significant mechanical properties will be discussed in the subsequent subsections, which are closely tied to the morphological analysis conducted prior.

### 3.1. Quasi-Static Nanoindentation

In an initial experimental study focused on the surface characterization of nanocomposite materials, the Oliver–Pharr method [42] was employed to assess the hardness (H), reduced elastic modulus (Er), and corresponding contact depth of the samples. 

More in detail, the nanoindentation tests employed in this study were performed under quasi-static loading conditions. This experimental setup follows the well-established Oliver–Pharr method, a robust framework for determining mechanical properties such as hardness and elastic modulus from the indentation load–displacement data. The quasi-static nature of the test ensures minimal dynamic effects, allowing for precise characterization of the material’s response. By adhering to this methodology, we have ensured that our results are accurate, reproducible, and consistent with current standards in nanoindentation analysis.

Specifically, nanoindentation hardness is calculated by dividing the maximum applied load (Pmax) by the indenter’s contact area (A). To determine the reduced elastic modulus, the unloading phase of the load–displacement curve is fitted with a power-law function, allowing for the extraction of stiffness. Following this, Er is computed by establishing a mathematical link between the unloading stiffness and the projected contact area under load. Importantly, this calculation accounts for elastic deformations in both the sample and the indenter. In fact, the reduced modulus (*Er*) is derived from the nanoindentation test using Sneddon’s formula [43]:

(11) $$\frac{1}{E_{r}}=\frac{\left(1-\nu^{2}\right)}{E}+\frac{\left(1-\nu_{i}^{2}\right)}{E_{i}}$$

In this formula, *E* and *ν* account the elastic modulus and Poisson’s ratio of the test material, while *E_i_* and *ν_i_* correspond to the elastic modulus and Poisson’s ratio of the indenter tip. 

**Figure 8:**
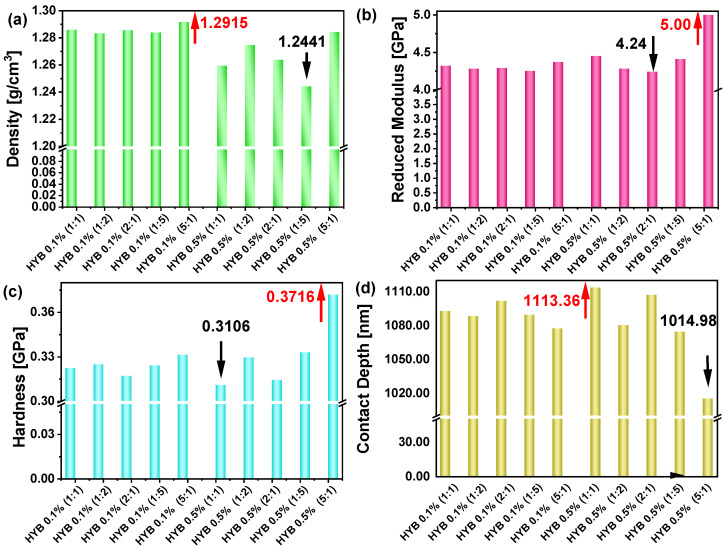
Density (**a**), reduced modulus (**b**), hardness (**c**), and contact depth (**d**) for all nanocomposites investigated in the present study. In each subplot, the red upward arrow indicates the maximum value for that property, with the numerical value displayed, while the black downward arrow marks the minimum value.

Figure 8 reports the experimental outcomes for four different properties of hybrid materials at varying concentrations and proportions. The properties include density in (a), reduced modulus in (b), hardness in (c), and contact depth in (d) for different proportions of hybrid materials, specifically 0.1% and 0.5%, across different weight ratios of MWCNTs:GNs (i.e., 1:1, 1:2, 2:1, 1:5, 5:1). Each property is represented in a separate subplot, while all measured values are summarized in Table 2.

Density provides an indication of the mass per unit volume of the material. The density values for all hybrid materials examined in this study remain quite consistent, indicating that the composition changes only slightly affect the overall mass distribution.

In fact, for both 0.1% and 0.5% formulation materials, the variation in density is minimal, ranging from 1.24 for HYB 0.5% (1:5) to 1.29 g/cm^3^ for HYB 0.1% (5:1), which suggests that changes in the hybrid ratio do not dramatically alter the density of the materials. This consistency can be attributed not only to the similar true densities of the two fillers—2.25 g/cm^3^ for GNs and 2.26 g/cm^3^ for CNTs—but also to the high-quality preparation and uniform dispersion of the materials within the epoxy matrix. The similar densities of CNTs and GNs mean that, even with variations in their relative proportions, the overall density of each composite formulation remains largely unchanged. The comparable densities of these carbon-based nanomaterials create a balanced system within the resin, reducing the impact of filler concentration on the composite’s density.

Furthermore, the careful preparation of these composites played a significant role in achieving homogeneity and minimizing density variations. The meticulous dispersion of both CNTs and GNs throughout the epoxy by ultrasonication for at least 30 min ensures a consistent material structure, where any variations in filler concentration do not lead to significant density fluctuations. This level of uniformity is essential for producing composites with predictable and reproducible mechanical properties, further underscoring the effectiveness of the preparation methods used in this study.

The reduced modulus represents the elastic response of the material under indentation and provides an understanding of its stiffness when considering both the material and the indenter’s properties. Higher reduced modulus values imply stiffer materials.

The lowest value is observed for HYB 0.1% (1:1) at 4.24 GPa, suggesting this composition has a relatively lower stiffness compared to the other samples. The highest value is for HYB 0.5% (5:1), reaching 5.0 GPa, indicating this composition has the highest stiffness among the tested materials.

The materials with 0.5% concentration tend to show higher stiffness compared to those with 0.1%, as evidenced by the higher reduced modulus values in the 0.5% set.

Hardness is a measure of a material’s resistance to localized plastic deformation. A higher hardness value indicates a material that is more resistant to irreversible deformation.

The lowest hardness value is observed for HYB 0.5% (1:1) at 0.3106 GPa, indicating this material is the softest in terms of its resistance to localized deformation. The highest hardness value is for HTB 0.5% (5:1) at 0.3716 GPa, suggesting this composition is the hardest material in the set, with the greatest resistance to deformation.

The hardness values show a slight increase as the concentration of the hybrid material increases from 0.1% to 0.5%. Additionally, increasing the ratio from 1:1 to 5:1 tends generally to increase the hardness, with the highest value recorded for the 5:1 ratio in the 0.5% concentration. This indicates that both the concentration and the ratio of the hybrid materials affect the hardness, with higher ratios and concentrations leading to increased hardness.

Contact depth is a measure of the penetration depth of the indenter during the hardness test. A lower contact depth indicates a harder material, while a higher contact depth suggests a softer material.

The lowest value is observed for HYB 0.5% (5:1), with a value of 1014.98 nm. This suggests this material has the highest resistance to indentation, correlating with higher hardness and stiffness. The highest value is observed for HYB 0.5% (1:1) at 1113.36 nm, indicating that this composition is the softest among the tested samples and offers less resistance to indentation. More generally, the contact depth decreases as the proportion of one component increases (from 1:1 to 5:1) for both the 0.1% and 0.5% concentrations. This suggests that materials with higher hybrid ratios or increased concentration have higher resistance to the indenter’s displacement, which correlates with increased hardness.

In summary, the HYB 0.5% (5:1) sample, which has the highest carbon nanotube concentration, shows the best mechanical properties compared to those measured for other formulations. More in general, the increase in the ratio of multi-walled carbon nanotubes (MWCNTs) to graphene nanoplatelets (GNs), as deeply investigated in the (1:1) and (5:1) MWCNTs:GNs compositions, can be attributed to several contributing factors. MWCNTs are known for their high stiffness, tensile strength, and excellent load-bearing capacity. When their concentration increases relative to GNs, they contribute more significantly to reinforcing the material. This reinforcement creates a stiffer matrix that can resist greater applied loads, resulting in reduced penetration during nanoindentation, which directly correlates to a shallower contact depth. Furthermore, as the ratio of MWCNTs increases, the load during indentation is distributed more evenly across the material due to the effective load transfer properties of MWCNTs. This load distribution limits localized deformation and reduces the material’s ability to indent, thereby decreasing the contact depth.

Again, the MWCNTs may establish stronger interfacial bonding with the surrounding matrix compared to GNs. This enhanced bonding increases the rigidity and reduces the plastic deformation under applied forces. A higher content of MWCNTs leads to a more robust network that limits the depth of indentation and results in improved hardness. This last property is directly related to a material’s resistance to permanent deformation. Since MWCNTs possess higher intrinsic hardness and stiffness compared to GNs, increasing their weight percentage (wt%) enhances the overall hardness of the composite. The denser and more rigid MWCNT network makes it more difficult for the indenter to penetrate deeply, explaining the increase in hardness.

**Figure 9:**
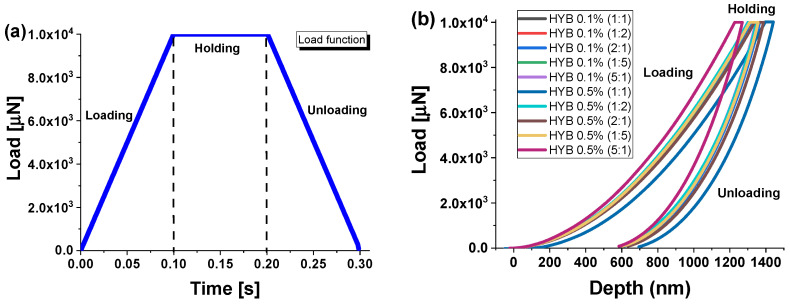
In (**a**), a trapezoidal load function utilized for nanomechanical characterization. In (**b**), the force versus displacement curves for all the samples analyzed.

Figure 9 shows the average load–displacement curves (from 49 tests), illustrating the varying degrees of indenter penetration into the composite samples. These curves provide insight into the material’s response under different loading conditions, revealing how the indenter interacts with each sample based on its mechanical properties.

Applying a trapezoidal load function with a peak force of 10,000 µN (see Figure 9a) caused the indenter to penetrate more than 700 nm into the material (light blue curve, sample HYB 0.5% (1:1)). This deeper displacement allows for a more detailed analysis of the material’s mechanical behavior, including both its elastic modulus and hardness. Physically, a greater penetration depth provides insight into how the internal structure of the composite accommodates the applied stress, revealing characteristics such as material ductility, stiffness, and resistance to strain. The reduced maximum displacement observed in the curves for the HYB 0.5% (5:1) sample (purple curve) indicates a higher resistance to indenter movement. This suggests that this composite has a more robust morphological network inside the matrix, which is better at distributing the applied force throughout its structure. Such behavior implies a stiffer material, with stronger interfacial bonding between its components, thereby enhancing its overall mechanical properties compared to the other samples examined, highlighting its potential for applications requiring high mechanical strength.

These results are clearly consistent with the contact depth measurements discussed earlier, as the HYB 0.5% (1:1) and HYB 0.5% (5:1) samples showed the maximum and minimum values, respectively, reflecting these curves as the load varies.

**Figure 10:**
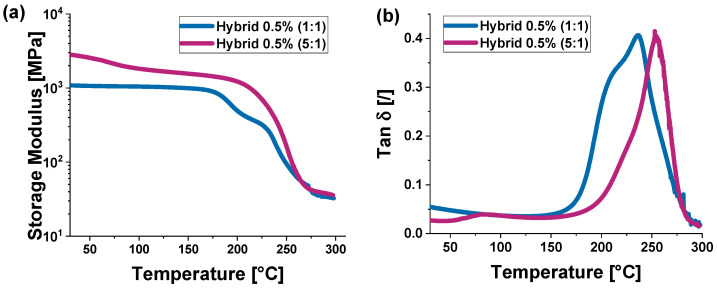
DMA curves for the HYB 0.5% (1:1) and HYB 0.1% (5:1) samples: (**a**) storage modulus vs. temperature; (**b**) Tan δ vs. temperature.

It is worth highlighting how the outcomes from the nanomechanical characterization closely correspond with the experimental results obtained from the Dynamic Mechanical Analysis (DMA) performed in our earlier research [29]. For clarity and ease of comparison, these findings are referenced here. Figure 10 illustrates the storage modulus and Tan δ as functions of temperature for the HYB 0.5% (1:1) and HYB 0.1% (5:1) samples, shown in panels (a) and (b), respectively.

The storage modulus is a measure of the strain rate sensitivity of a material and is commonly used in viscoelastic analysis to evaluate mechanical properties.

With reference to this property (panel a), both curves (the purple curve represents the sample HYB 0.5% (5:1), while the light blue curve represents the sample HYB 0.5% (1:1)) exhibit a similar behavior: initially (at low temperatures), the storage modulus is high, indicating that the material is stiffer, but then gradually decreases as the temperature increases.

However, the purple curve is higher than the light blue one along most of the graph, meaning that the material with the 5:1 ratio has a higher storage modulus (stiffness) compared to the material with the 1:1 ratio.

The most significant decline starts after 130 °C, becoming particularly noticeable between 180 °C and 230 °C, with both materials losing stiffness as they approach higher temperatures.

Moreover, the area under both curves represents the amount of energy stored in the material as mechanical stiffness over the temperature range considered.

A larger area under a curve indicates that the material has an overall higher storage modulus over a wider temperature range. In this case, since the purple curve is generally above the blue one, it means that the material with the 5:1 ratio retains greater stiffness than the material with the 1:1 ratio over the temperature range.

In summary, the HYB 0.5% (5:1) sample has a higher resistance to deformation under mechanical stress and maintains its stiffness over a wider temperature range compared to the HYB 0.5% (1:1) sample. This could indicate that the formulation with the filler mix ratio of (5:1) is more suitable for applications where higher mechanical stiffness at elevated temperatures is required.

Differently, the Tan δ is the ratio between the loss modulus (viscous modulus) and the storage modulus (elastic modulus), and it indicates the damping capacity and viscoelastic behavior of a material. The higher the value of Tan δ, the greater the viscous component relative to the elastic one. Regarding this property, both materials exhibit similar behavior (panel b), with Tan δ remaining low at lower temperatures, indicating that the materials primarily behave elastically (rigid). However, as the temperature increases, Tan δ rises, reaching a maximum and then rapidly decreasing.

The curve for HYB 0.5% (1:1) (in light blue) shows a peak at a lower temperature compared to HYB 0.5% (5:1) curve (in purple), indicating that the mix ratio (1:1) of the sample has a lower Tg (glass transition temperature) than the combination ratio (5:1). Therefore, the sample with the mix ratio (1:1) appears to undergo its glass transition at a lower temperature, which may suggest it is more flexible or less rigid compared to the sample with the mix ratio (5:1), which has a higher Tg, indicating that the sample with the mix ratio (5:1) sample is more rigid or thermally stable. Furthermore, the HYB 0.5% (1:1), which became more flexible earlier (it has a lower Tg), has a lower capacity to absorb energy compared to HYB 0.5% (5:1). 

After the peak, the rapid drop in Tan δ indicates that both materials have lost much of their rigidity and behave more viscoelastically at higher temperatures.

In any case, before the peak, for both HYB 0.5% (1:1) and HYB 0.5% (5:1) samples, the areas under the curves are relatively small. This indicates that in this phase (at lower temperatures), both materials have a low capacity for energy dissipation and behave mainly in an elastic (rigid) manner. 

The area under the curve of the Tan δ peak is an indicator of the amount of energy the material can absorb and dissipate during this transition.

HYB 0.5% (1:1) has a larger overall area under the curve around the peak. This suggests that the sample with the mix ratio (1:1) can dissipate more energy during the glass transition but also experiences a greater loss in rigidity compared to the sample with the mix ratio (5:1) in this phase. After the peak, the areas under the curves decrease rapidly, indicating that the material has lost its rigidity and behaves more viscoelastically.

These results show a strong correlation with the nanoindentation data. 

About this, a higher contact depth for HYB 0.5% (1:1) (1113.36 nm) indicates that this material is softer as the indenter penetrates deeper into the surface. This softness is also reflected in the lower storage modulus (graph a of Figure 10), confirming that the material offers less resistance to deformation.

Conversely, HYB 0.5% (5:1) shows a lower contact depth (1014.98 nm), indicating it is harder and more resistant to indentation. This correlates with the higher storage modulus observed in the same graph. 

The HYB 0.5% (1:1) sample has a lower hardness (0.3106 GPa), which is consistent with the lower storage modulus. The HYB 0.5% (5:1) sample, on the other hand, shows greater hardness (0.3716 GPa), aligning with its higher storage modulus generally above that of HYB 0.5% (1:1) in the entire temperature range.

This influence of concentrations and weight ratios on the mechanical properties will be further investigated through a Design of Experiments (DOE) and Response Surface Methodology (RSM) approaches in the following section. These two formulations will also undergo a numerical study using software based on the finite element method (FEM) for deeper insights.

### 3.2. Design of Experiment (DoE) for the Nanomechanical Properties

The Design of Experiments (DoE) methodology results in the creation of both the Dex Scatter Plot (DSP) and the Main Factor Plot (MFP). The DSP provides a visual representation of the experimental data, showcasing the relationship between the dependent variable and the various controllable input factors. It helps identify the most influential variables and how they affect the performance function (P.F.), whether improving or worsening it. Complementing this, the MFP is used to compare the average responses for different input factors, offering a clear view of how each factor impacts the system by analyzing the slopes or lack thereof in the plotted data: a horizontal line indicates no effect, while a slope indicates measurable influence.

**Figure 11:**
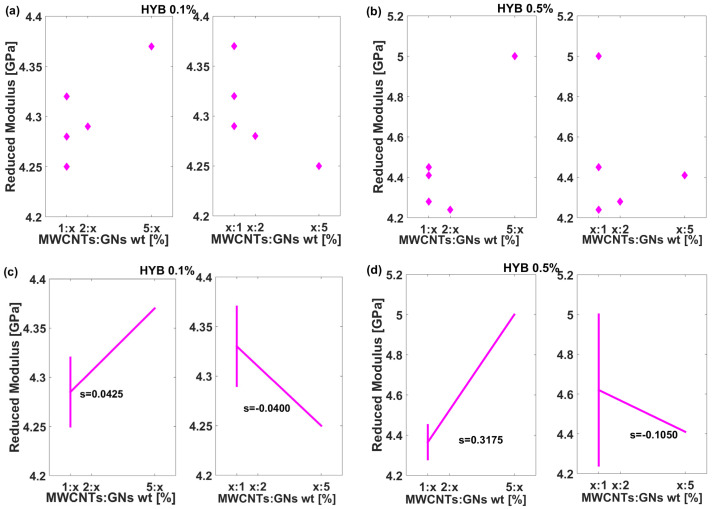
Dex Scatter Plot (DSP) and Main Factor Plot (MFP) for the experimental data of the reduced modulus for HYB 0.1% (1:1) in panels (**a**,**c**) and HYB 0.1% (5:1) in panels (**b**,**d**), respectively.

The results shown in Figure 11 for both the DSP (a and b) and MFP (c and d) highlight variations in the behavior of the Reduced Modulus (in GPa) for two different composite formulations, HYB 0.1% (MWCNTs:GNs) and HYB 0.5% (MWCNTs:GNs), which, in the graphs, have been shortened to HYB 0.1% and HYB 0.5%, as the mix weight ratio of multi-walled carbon nanotubes (MWCNTs) to graphene nanosheets (GNs) is adjusted.

More in detail, the scatter plot shows (panels a and b) that for the HYB 0.1% (MWCNTs:GNs) formulation, the reduced modulus values exhibit minor variations with changes in the MWCNTs:GNs mix weight ratio. The values tend to cluster around 4.25–4.35 GPa across different mix ratios (1, 2, 5). In contrast, the HYB 0.5% (MWCNTs:GNs) formulation shows larger shifts in the reduced modulus, with values ranging from around 4.2 GPa to approximately 5 GPa, indicating a more pronounced response to changes in the filler weight ratio. This is more evident from the analysis of the MFP plots of Figure 11c,d. For both variational parameters, namely, the weight content of MWCNTs and GNs, the slopes of the MFP are always greater in the case of the HYB 0.5% (MWCNTs:GNs) formulation compared to those estimated for the HYB 0.1% (MWCNTs:GNs) formulation. In particular, for the first subplot (c, left part), the slope is 0.0425, showing a slight increase in modulus with increasing MWCNT content. 

However, in the second subplot (c, right part), the slope is −0.0400, indicating a decrease in modulus at higher GN weight ratios. This suggests that, at lower filler content (0.1%), there is a minor but opposite trend in modulus depending on the ratio of MWCNTs to GNs. For the higher filler mix concentration of 0.5 wt%, the slopes are much more significant. The first subplot (d, left part) has a steep positive slope of 0.3175, indicating a strong increase in modulus as the MWCNT content increases. Conversely, the second subplot (d, right part) shows a negative slope of −0.1050, although not as steep as in the first case. This suggests that while the modulus increases substantially with more MWCNTs, it decreases as GNs dominate the hybrid filler.

These trends align with physical principles related to load transfer and interfacial strength in nanocomposites. At a lower filler mix content of 0.1 wt%, the individual contributions of MWCNTs and GNs are limited, so their impact on modulus is modest and largely independent of the specific filler ratio. As filler content increases up to 0.5 wt%, interfacial interactions between the matrix and fillers become more significant. MWCNTs, with their high aspect ratio and mechanical strength, likely enhance modulus more effectively due to better load transfer, whereas GNs may not contribute as strongly to modulus but rather introduce sites that may slightly reduce stiffness. This behavior is consistent with the observed positive slope for MWCNTs and the negative slope for GNs at higher filler content. The findings underscore how both filler type and concentration need to be optimized to achieve the desired mechanical properties in hybrid nanocomposites.

**Figure 12:**
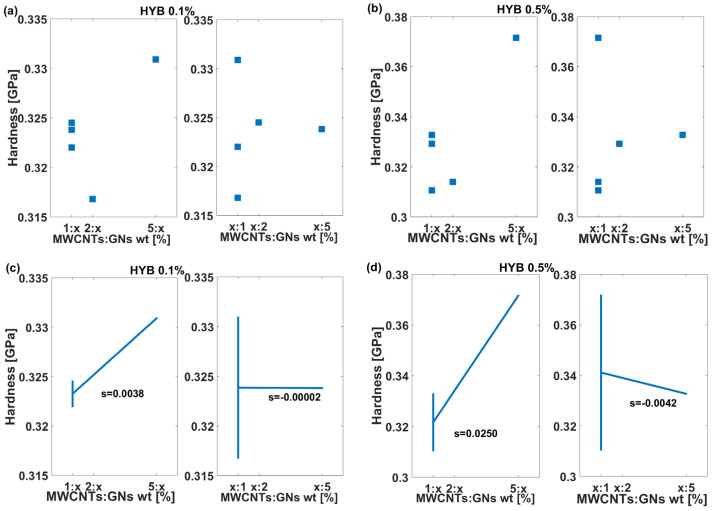
Dex Scatter Plot (DSP) and Main Factor Plot (MFP) for the experimental data of the hardness for HYB 0.1% (1:1) in panels (**a**,**c**) and HYB 0.1% (5:1) in panels (**b**,**d**), respectively.

The results shown in the Dex Scatter Plot (a and b) and Main Factor Plot (c and d) of Figure 12 present the hardness (measured in GPa) of composite samples belonging to the two formulations: HYB 0.1% (MWCNTs:GNs) and HYB 0.5% (MWCNTs:GNs), which, in the graphs, have been shortened to HYB 0.1% and HYB 0.5%. The goal is to analyze how varying the weight ratios of MWCNTs:GNs affects the hardness of the material and to highlight the differences in behavior between the two formulations. In particular, for the HYB 0.1% (MWCNTs:GNs) formulation, the hardness values fall within a narrow range between approximately 0.315 and 0.335 GPa. There is some slight variability depending on the MWCNT weight ratio, while changes in GN content do not significantly impact hardness. In contrast, the Dex Scatter Plot (b) for HYB 0.5% (MWCNTs:GNs) shows a wider range of hardness values, from around 0.30 to 0.38 GPa. This indicates a more significant response of the composite to the different filler weight ratios. These aspects are better highlighted by the graphical analysis of the MFP plots (c and d). In the Main Factor Plot for the HYB 0.1% (MWCNTs:GNs) formulation (c, left), the slope is positive but very shallow, with a value of 0.0038, indicating only a slight increase in hardness as MWCNT content increases. In the second subplot (c, right), the slope is virtually zero (−0.00002), indicating no significant change in hardness as the GN weight ratio increases. This suggests that at 0.1% filler mix content, the hybrid system has a very limited impact on hardness. For the HYB 0.5% (MWCNTs:GNs) formulation, the effect is much more pronounced. The first subplot (d, left) has a steeper positive slope of 0.0250, suggesting a strong increase in hardness with increasing MWCNT content. In contrast, the second subplot (d, right) has a negative slope of −0.0042, showing that as the GNs become the dominant filler component, the hardness decreases slightly.

In summary, the MWCNT weight ratio plays a more substantial role in influencing hardness compared to the influence of GNs. The positive slope in both cases shows that adding MWCNTs generally enhances the hardness while increasing GNs can lead to a slight decrease. The hardness values are much more variable at the higher filler mix content of 0.5%, highlighting a stronger interaction between the filler components and the matrix. This suggests that at higher concentrations, the hybrid filler system is more effective at altering the mechanical properties of the composite material.

To summarize, these findings underscore the dominant role of MWCNTs in enhancing hardness, particularly at higher concentrations. The higher aspect ratio and inherent stiffness of MWCNTs likely contribute to greater load transfer within the matrix, thus reinforcing the composite and increasing hardness more effectively than GNs. Conversely, as GN content increases, the decrease in hardness could be due to the relatively flat, platelet-like structure of GNs, which may not support the matrix as effectively under stress, leading to a slight reduction in hardness. Overall, at a higher filler mix concentration of 0.5 wt%, the hybrid system shows a much stronger impact on hardness, demonstrating that the interaction between the fillers and the matrix becomes more influential. This suggests that optimizing MWCNT content is key to maximizing hardness in hybrid composites, while GN content needs careful adjustment to prevent any reduction in mechanical reinforcement. This knowledge can guide the design of composites with tailored mechanical properties, making the hybrid filler system particularly valuable in applications requiring enhanced hardness and durability.

**Figure 13:**
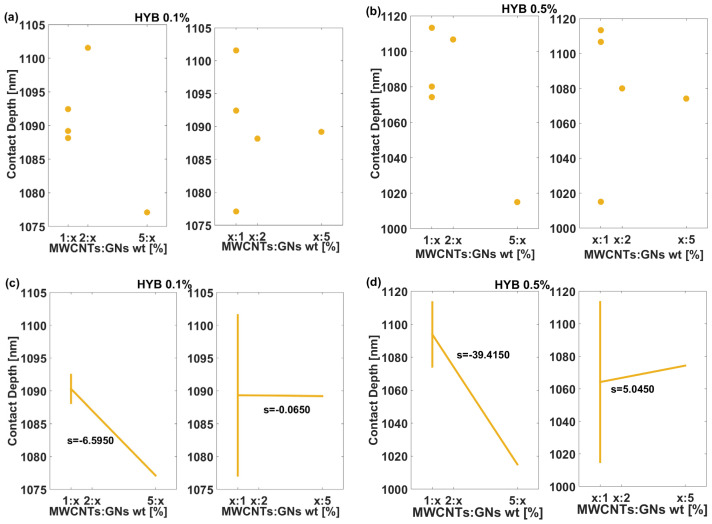
Dex Scatter Plot (DSP) and Main Factor Plot (MFP) for the experimental data of the contact depth for HYB 0.1% (1:1) in panels (**a**,**c**) and HYB 0.1% (5:1) in panels (**b**,**d**), respectively.

To conclude this statistical analysis, Figure 13 shows how the contact depth (measured in nm) for the two different composite formulations, HYB 0.1% (MWCNTs:GNs) and HYB 0.5% (MWCNTs:GNs), which, in the graphs, have been shortened to HYB 0.1% and HYB 0.5%, is influenced by varying the weight ratios of MWCNTs:GNs. The data are presented through both the Dex Scatter Plot (a and b) and the Main Factor Plot (c and d). These plots allow for an analysis of how the weight ratio of the filler content affects the contact depth and, once again, focus on the differences between the two formulations.

For HYB 0.1% (MWCNTs:GNs), the contact depth values are relatively close, ranging between 1080 and 1105 nm, thus indicating that at lower filler mix content (0.1 wt%), the MWCNT ratio has a limited effect on the contact depth (a, left). A negligible influence is observed with varying GN content in the weight ratio with MWCNTs (a, right). The HYB 0.5% (MWCNTs:GNs) formulation exhibits a wider range of contact depth values, from around 1000 nm to 1120 nm, indicating a more pronounced response of the composite as the MWCNT ratio changes. The scatter plot suggests a greater variability in the contact depth at higher filler content, which reflects the stronger influence of the filler components on this mechanical property. In the Main Factor Plot for the HYB 0.1% (MWCNTs:GNs) formulation, the first subplot (c, left part) shows a moderate negative slope (s = −6.5950) for the MWCNT ratio, indicating a decrease in contact depth with increasing MWCNT content. The second subplot (c, right part) presents a very shallow negative slope (s = −0.0650), indicating that increasing the GN content has virtually no effect on contact depth at this filler concentration. For the HYB 0.5% (MWCNTs:GNs) formulation, the Main Factor Plot shows more significant changes. The slope for the first subplot (d, left) is quite steep (s = −39.4150), revealing a substantial reduction in contact depth as the MWCNT content increases. In contrast, the second subplot (d, right) has a positive slope (s = 5.0450), suggesting that increasing GNs leads to a slight increase in contact depth.

The slopes for HYB 0.5% (MWCNTs:GNs) are significantly steeper than those for HYB 0.1% (MWCNTs:GNs), especially for the MWCNT-rich ratios. This indicates that the contact depth is far more sensitive to changes in filler ratios at higher filler contents. Regardless of the formulation (HYB 0.1% (MWCNTs:GNs) or HYB 0.5% (MWCNTs:GNs)), the steep negative slopes suggest that adding more MWCNTs in the filler weight ratio significantly reduces contact depth, possibly due to an increase in stiffness or resistance to indentation.

In summary, these findings indicate that contact depth is far more responsive to filler mix ratio changes at higher concentrations (HYB 0.5% (MWCNTs:GNs)), especially with MWCNT-rich compositions. The steep negative slopes for MWCNTs suggest that increasing MWCNT content contributes to higher stiffness and resistance to indentation, effectively reducing contact depth due to MWCNTs’ mechanical rigidity and load-bearing capacity. On the other hand, the GN content appears to counteract this effect slightly, potentially due to its planar structure, which may introduce microstructural adjustments that allow for a minimal increase in contact depth. In conclusion, the analysis underscores the impact of filler optimization on mechanical properties in composite materials, as MWCNTs significantly improve stiffness at higher concentrations, while GNs introduce minor flexibility. These insights are valuable for designing composites tailored to specific mechanical performance requirements, especially where contact depth and indentation resistance are critical factors.

### 3.3. Response Surface Methodology (RSM)

**Figure 14:**
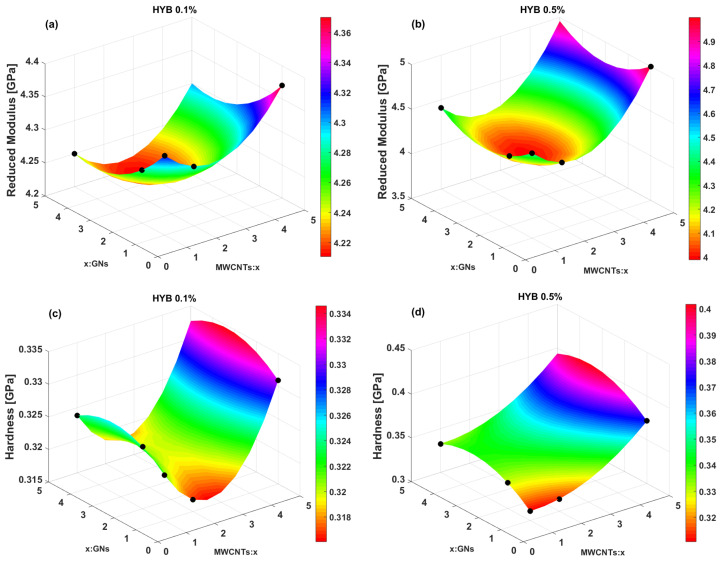

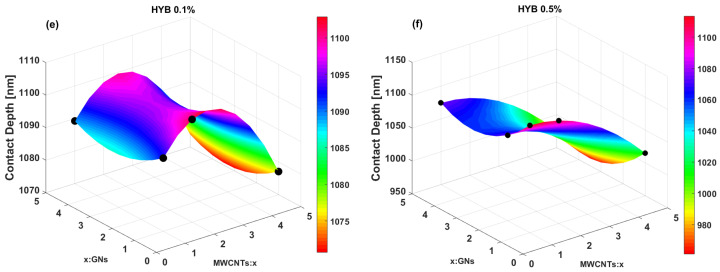
Response surface plots representing the mechanical properties of the two HYB formulations. Subplots (**a**,**b**) display the reduced modulus for the 0.1% and 0.5% HYB compositions, respectively. Subplots (**c**,**d**) illustrate the hardness values for each formulation, while (**e**,**f**) depict the contact depth. Experimental data points are marked with black dots, demonstrating the fit of the surface to the measured values. The color bar in each subplot shows the range of variability for each property.

The statistical technique of Response Surface Methodology (RSM) is employed in our study to derive an analytical equation (Equation (10) previously introduced) that captures the relationship between the nanomechanical properties—such as reduced modulus, hardness, and contact depth—obtained experimentally through nanoindentation tests. These properties are analyzed in relation to the varying weight ratios of multi-walled carbon nanotubes (MWCNTs/x) and graphene nanosheets (x/GNs) in the two composite formulations, HYB 0.1% (MWCNTs:GNs) and HYB 0.5% (MWCNTs:GNs), which, in the graphs, have been shortened to HYB 0.1% and HYB 0.5%. This approach provides valuable insights into optimizing the composite structure for enhanced performance. Figure 14 showcases the graphical trend of the estimated response surfaces for the various mechanical properties. Specifically, subplots (a) and (b) show the response surfaces for the reduced modulus of the HYB 0.1% (MWCNTs:GNs) and HYB 0.5% (MWCNTs:GNs) formulations, respectively. In (c) and (d), the surfaces represent the hardness for the same formulations, while (e) and (f) depict the contact depth. The color bar in each subplot shows the range of variability for each property. The experimental points are displayed as black dots on each predicted surface to visually assess the accuracy of the RSM models. All coefficients of the RSM are stated in Table 3.

Here is a breakdown of the results for each property. With reference to the HYB 0.1% (MWCNTs:GNs) formulation (panel a), the reduced modulus varies between 4.22 GPa and 4.36 GPa. The surface shows a concave pattern, with a moderate increase in modulus with increasing MWCNT weight content. The reduced modulus for HYB 0.5% (MWCNTs:GNs) (panel b) ranges from 4.4 GPa to 4.9 GPa, which is significantly higher compared to HYB 0.1% (MWCNTs:GNs). The surface response is more dynamic, showing a steep rise in modulus at higher MWCNT contents, suggesting a substantial improvement in mechanical properties with the increase in this filler content.

For both formulations, the hardness values are relatively close (0.31 to 0.34 GPa for HYB 0.1% (MWCNTs:GNs), see panel c, and 0.30 GPa to 0.40 GPa for HYB 0.5% (MWCNTs:GNs), see panel d), but they exhibit distinct surface patterns. The HYB 0.1% (MWCNTs:GNs) sample shows a dip in hardness at intermediate concentrations of GNs and MWCNTs, while HYB 0.5% (MWCNTs:GNs) shows a more uniform increase in hardness with increasing concentrations.

For HYB 0.1% (MWCNTs:GNs), the contact depth (panel e) varies more dramatically across the range of variability for GNs and MWCNTs, with higher contact depths (weaker resistance) at intermediate levels of both fillers. The contact depth ranges from approximately 1070 nm to 1110 nm. Contrarily, HYB 0.5% (MWCNTs:GNs) shows more stability in contact depth (panel f), with the depths generally decreasing (higher resistance to indentation) as the filler concentrations increase. The contact depth spans from around 950 nm to 1150 nm. This indicates that HYB 0.5% (MWCNTs:GNs) has more consistent mechanical behavior under load, which is likely due to better reinforcement provided by the higher filler content.

In summary, the HYB 0.5% (MWCNTs:GNs) formulation generally shows higher modulus and hardness and lower contact depths, which is consistent with the idea that adding more fillers like MWCNTs strengthens the material and enhances its mechanical properties. The response surfaces for HYB 0.5% (MWCNTs:GNs) are smoother, suggesting more uniform behavior as a result of the higher filler content.

Finally, it is worth emphasizing that in all cases, there is a clear alignment between the estimated surfaces and the actual measurements, indicating that the response surfaces effectively capture the experimental trends. This close match reflects the robustness of the regression model (RSM) used to generate these surfaces, suggesting that the model accurately predicts the relationship between the material’s mechanical properties and the conditioning parameters, such as the concentration of fillers. The strong correlation between the experimental data and the R.S. also emphasizes the reliability of the model in capturing the physical behavior of the material, specifically in the complex domain of nanocomposites, where interactions between components can significantly influence performance. This ability to estimate mechanical properties, including reduced modulus, hardness, and contact depth, based on input variables demonstrates the utility of RSM in optimizing material design and performance predictions. Such modeling is particularly valuable in the development of advanced materials like nanocomposites, where the precise control of microstructural parameters is key to achieving desired properties.

### 3.4. Multhyphisics Simulation Study: Results

The finite element (FE) method is a highly regarded and effective approach for analyzing the mechanical behavior of materials under various conditions. In this study, an integrated approach combining experimental data with FE simulations was applied, allowing for a direct comparison between the simulated model predictions and the actual experimental results.

After validating the FE model, it served as a foundation for exploring additional mechanical properties in more detail. The simulations were then tailored specifically to two formulations, HYB 0.5% (1:1) and HYB 0.5% (5:1), selected for their distinctly different mechanical responses as identified in preliminary experimental nanoindentation analyses.

#### 3.4.1. Validation Model

**Figure 15:**
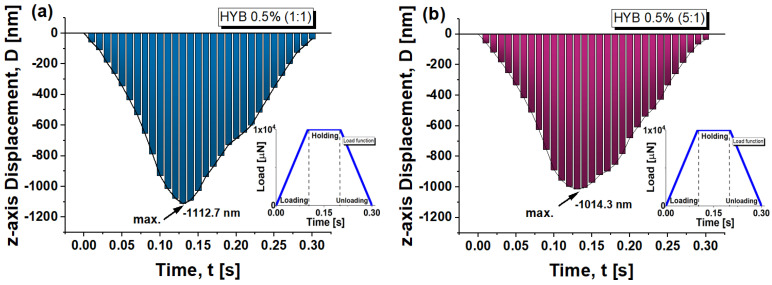
The z-axis displacement versus the entire time interval [0, 0.3] s for the HYB 0.5% (1:1) sample in (**a**) and the HYB 0.5% (5:1) sample in (**b**).

Figure 15 displays the z-axis displacement, D, over time for two material formulations, HYB 0.5% (1:1) in subplot (a) and HYB 0.5% (5:1) in subplot (b), in response to a trapezoidal loading function. The trapezoidal function, depicted in the insets, includes a loading phase that ramps up to a peak load of approximately 10,000 µN, followed by a brief holding phase, and then an unloading phase back to zero load.

The graphs offer a comprehensive understanding of the material’s mechanical response during the nanoindentation process. They highlight how the material deforms under load, showcasing its displacement and recovery characteristics throughout both the loading and unloading phases. This detailed depiction allows for a clearer analysis of the differences in behavior between the two samples, shedding light on their stiffness, compliance, and overall performance under applied force. In both cases, the overall curve shape appears to be triangular or cone-like, indicating the loading and unloading phases of the indentation experiment. In both graphs, the maximum z-displacement (*D*) occurs around the middle of the time interval, roughly 0.13 s, and shows the material reaching a maximum indentation depth.

The time duration for reaching maximum displacement is the same for both samples (~0.13 s), but the amplitude of the displacement differs, showing distinct mechanical behavior. The HYB 0.5% (1:1) sample has a larger deformation window. The HYB 0.5% (1:1) sample (subplot a) reaches a maximum displacement of approximately −1112.7 nm, whereas the HYB 0.5% (5:1) sample (subplot b) reaches a slightly lower maximum displacement around −1014.3 nm.

This confirms that the HYB 0.5% (1:1) sample is more prone to deformation compared to the HYB 0.5% (5:1), leading to more depth penetration during the test.

The increase in the ratio of multi-walled carbon nanotubes (MWCNTs) to graphene nanosheets (GNs), as it is in the 5:1 compared to the 1:1 composition, reduces the contact depth and improves the hardness of the resulting material.

In brief, for both formulations, the displacement increases during loading and decreases during unloading, with residual deformation present after unloading, implying permanent deformation.

The HYB 0.5% (5:1) formulation shows better recovery from deformation, with less permanent displacement (−34.230 nm) after unloading compared to the HYB 0.5% (1:1) formulation, which, in turn, exhibits a deformation value of 35.824 nm.

Above all, this investigation of the z-displacement over time allows us to obtain a numerical evaluation of the contact depth measurement, which is critical for validating the accuracy of our numerical model by comparing it with experimental data obtained for this parameter. Ensuring that the simulated model achieves a similar maximum displacement allows us to validate the model’s accuracy in replicating real material behavior under similar loading conditions.

**Figure 16:**
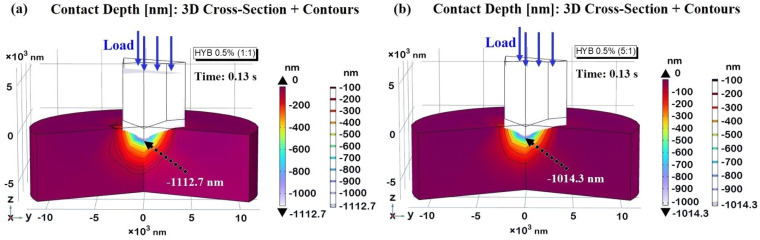
The contact depth measurements for HYB 0.5% (1:1) in (**a**) and HYB 0.5% (5:1) in (**b**) are shown at the time point t = 0.13 s, where the maximum contact depth occurs. The applied load is 10,000 µN. The contour lines emphasize the distinct indentation patterns for each sample.

In Figure 16, the maximum contact depth is shown at the critical time point of t = 0.13 s for the HYB 0.5% (1:1) and HYB 0.5% (5:1) samples in subplots (a) and (b), respectively. The calculated maximum contact depths are −1112.7 nm for HYB 0.5% (1:1) and −1014.3 nm for HYB 0.5% (5:1), closely matching the experimentally measured values (−1113.36 nm and −1014.98 nm, respectively). This close correlation between the simulated and experimental contact depths is crucial, as it attests to the robustness of the adopted numerical model, confirming its capacity to faithfully replicate the complex mechanical response of each formulation under nanoindentation.

The 3D cross-sectional views presented in Figure 16 add another dimension of analysis, displaying contour lines that represent the extent of penetration of the indenter. These contours highlight the distinct depth profiles between the two formulations and offer a visual interpretation of the varying degrees of z-displacement due to differences in hardness. By illustrating the depth gradients across the contact region, these contour lines emphasize how the model captures material-specific responses to indentation loading, further validating the accuracy and reliability of our simulation.

**Figure 17:**
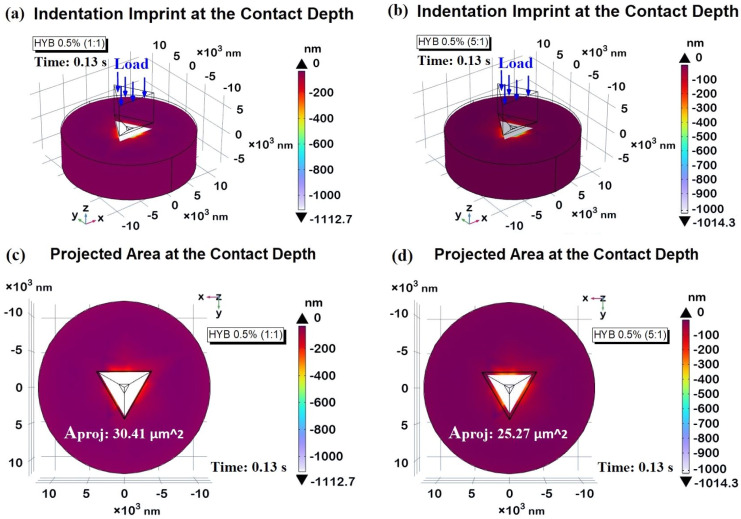
Indentation imprint at maximum contact depth for evaluating projected areas (Aproj) under an applied load of 10,000 µN. Panels (**a**,**b**) show the 3D views for the HYB 0.5% (1:1) and HYB 0.5% (5:1) samples, respectively, with the corresponding 2D top views displayed in (**c**,**d**).

The variation in contact depths between the HYB 0.5% (1:1) and HYB 0.5% (5:1) samples can be directly linked to their hardness values, a key property governing resistance to deformation. Harder materials, like HYB 0.5% (5:1), show a smaller contact depth and a shallower contour profile, while softer materials, such as HYB 0.5% (1:1), exhibit deeper penetration contours. The HYB 0.5% (5:1) sample, characterized by its higher hardness, has consistently lower z-displacement and shallower contour gradients. This reduced indentation depth reflects the material’s capacity to resist deformation more effectively, as its hardness enables it to withstand the applied load without significant penetration. The shallow contours in the 3D cross-section emphasize this resistance, showing a smooth and relatively shallow penetration profile. Materials with this level of hardness are well-suited for applications where high resistance to wear and minimal surface deformation are required, as they can maintain structural integrity under stress. The HYB 0.5% (1:1) sample, on the other hand, exhibits a greater z-displacement and more pronounced contour lines, indicative of deeper penetration. With its lower hardness, this material offers less resistance to the indenter, allowing for more substantial plastic deformation. The increased depth and sharper contours in the 3D view illustrate this behavior, showing a material that more readily accommodates the applied force, resulting in a deeper indentation. This property may be beneficial for applications that prioritize energy absorption or toughness over rigidity, as the material can undergo more substantial deformation under load. Figure 17 presents a three-dimensional representation of the indentation imprint at the peak contact depth (*h_i_*), specifically measuring −1112.7 nm and −1014.3 nm for the HYB 0.5% (1:1) sample and the HYB 0.5% (5:1) sample, illustrated in parts a) and b), respectively. The accompanying two-dimensional top views are depicted in parts c) and d) of the same figure. As anticipated and clearly demonstrated through the figure analysis, the projected areas vary in accordance with the function A_proj_ = *f*(*h_i_*). The tip of the indenter reaches a greater contact depth in the HYB 0.5% (1:1) sample, resulting in a significantly larger projected area compared to the HYB 0.5% (5:1) sample. Utilizing Equation (4), the computed area values are A_proj_ (HYB 0.5% (1:1)) = 30.41 µm^2^ and A_proj_ (HYB 0.5% (5:1)) = 25.27 µm^2^, respectively. This information is critical for calculating the hardness (*H*) of the material, as the indentation imprint serves as a fundamental parameter alongside the maximum applied load value (10,000 µN), in accordance with the established hardness equation presented as Equation (2).

The calculated values were 0.3290 GPa for HYB 0.5% (1:1) and 0.3960 GPa for HYB 0.5% (5:1), closely aligning with the values measured experimentally. These numerical results are compiled and compared with the experimental data in Table 4, which illustrates the percentage difference between the two data sets.

#### 3.4.2. z-Axis Displacement: Depth Rate and Insights into Material Thickness

Once the model has been validated and its accuracy in representing the fundamental mechanical properties of each formulation is confirmed, it becomes a robust tool for predicting material behavior in further investigations and provides valuable insights into the nanoindentation profile of each material.

**Figure 18:**
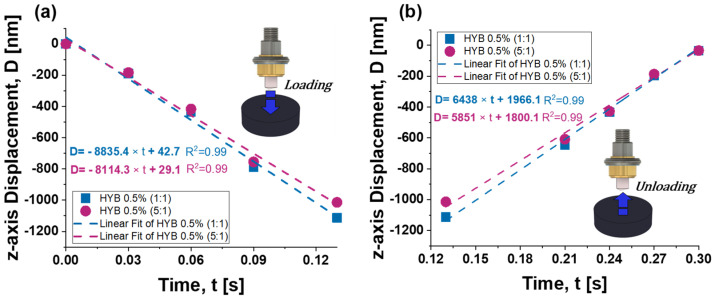
The z-axis displacement (D) over time (T) during the loading (**a**) and unloading phases (**b**) to evaluate the depth rate (DR) of the selected specimens as the slope of the linear fitting curve.

The depth rate (DR) is quantified as the rate of change or slope of z-displacement over time, representing how rapidly the indentation depth changes as time progresses. This measurement provides critical insights into the material’s response under an applied load, highlighting its deformation rate and resistance characteristics. By analyzing the depth rate, it is possible to evaluate how quickly a material undergoes displacement in the z-axis direction, offering a detailed understanding of both its hardness and viscoelastic properties. Figure 18 presents the depth rate findings for the two samples under study, assessed by the slope of the linear fit curve (R^2^ values very close to 1) representing the indenter’s displacement during both the loading (subplot a) and unloading phases (subplot b). The HYB 0.5 (5:1) sample, which displays higher hardness compared to the HYB 0.5 (1:1) sample, shows greater resistance to penetration by the indenter, resulting in a lower depth rate. The slopes calculated for the two samples during the loading time are 8835.4 and 8114.3, respectively.

During the unloading phase (from ~0.13 s to 0.30 s), the displacement recovery is less steep, and both samples demonstrate a near-symmetric recovery pattern. Both samples display good recovery behavior, although the HYB 0.5% (1:1) continues to show a faster unloading displacement (DR is equal to 6438 compared to 5851 for the 5:1 sample), indicating that it undergoes a faster strain recovery.

In brief, the HYB 0.5% (1:1) sample consistently shows a higher degree of compliance, larger deformation, and faster displacement rates during both loading and unloading compared to the HYB 0.5% (5:1) sample. This might suggest that the even distribution of carbon nanotubes and graphene nanosheets in the (1:1) sample leads to less reinforcement and more ductility, whereas the (5:1) sample (with higher MWCNT content) shows better stiffness and slower deformation rates.

In nanomechanical testing of composite materials, the z-axis displacement relative to material thickness offers valuable insights into the material’s deformation response under an applied load. Specifically, as the material’s thickness changes, the z-axis displacement captures the vertical shift of the surface as it reacts to the applied force. This correlation is closely tied to the material’s mechanical characteristics, including hardness, elasticity, and deformation resistance. The way the surface moves in relation to the applied load provides a deeper understanding of how the material withstands stress and recovers, thus shedding light on its overall mechanical properties.

**Figure 19:**
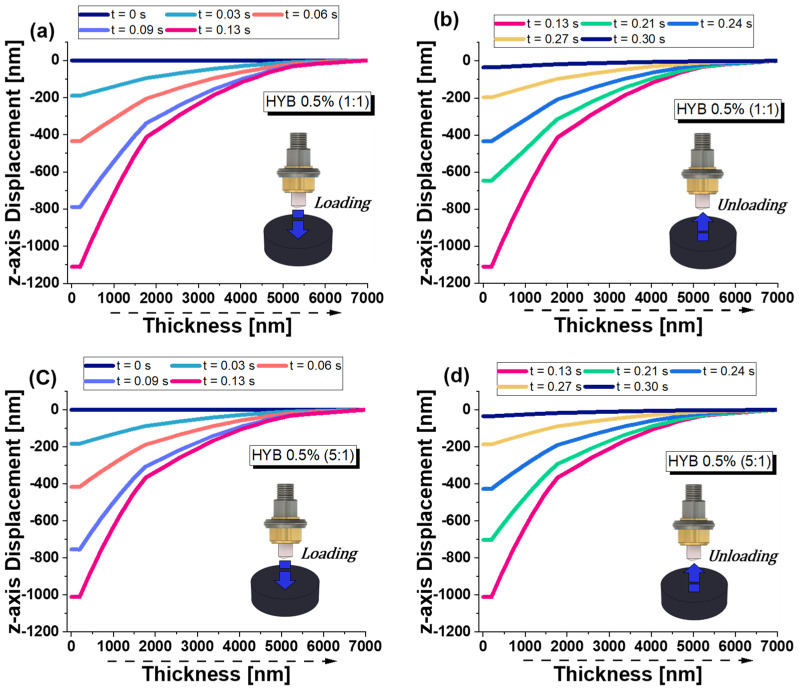
The z-axis displacement versus thickness at some selected instants of time for HYB 0.5% 1:1 during loading phase in (**a**) and unloading phase in (**b**). Same results for HYB 0.5% 5:1 sample during load phase in (**c**) and unloading phase in (**d**).

Figure 19 provides four graphs illustrating the z-axis displacement versus thickness for the two different formulations (HYB 0.5% (1:1) and HYB 0.5% (5:1)) under loading and unloading conditions at some specific time instants. Specifically, four time instants (0 s, 0.03 s, 0.06 s, and 0.09 s) were selected during the loading phase. During the holding phase, the time point at t = 0.13 s was analyzed, corresponding to the moment of maximum indentation depth. Finally, four time points (0.21 s, 0.24 s, 0.27 s, and 0.30 s) were chosen for the unloading phase.

During the loading phase for HYB 0.5% (1:1) in subplot (a) and for HYB 0.5% (5:1) (in subplot c), the graphics show a clear trend where the z-axis displacement increases progressively as the thickness increases.

There is a noticeable non-linear increase in displacement over time, with time increments of t = 0 s (blue), t = 0.03 s (orange), t = 0.06 s (green), t = 0.09 s (red), and t = 0.13 s (pink).

Displacement appears to saturate as the thickness approaches higher values (~5000 nm and above), indicating that the material is approaching a stable deformation point under loading conditions.

For the HYB 0.5% (5:1) formulation, the loading behavior follows a similar non-linear increase, as seen in the 1:1 ratio of MWCNTs:GNs. The displacement increases over time and with thickness, with the same time increments as in graph (a).

However, the magnitude of displacement is slightly lower than in the HYB 0.5% (1:1) formulation, which suggests that the 5:1 ratio formulation is stiffer and more resistant to deformation under the same loading conditions.

This lower displacement suggests that altering the composition to 5:1 improves the material’s ability to withstand deformation, thus indicating a stiffer and more resilient material.

The graphics (b) and (d) of Figure 19 display the unloading phase for the same HYB 0.5% (1:1) formulation and for HYB 0.5% (5:1), respectively.

The unloading behaviors mirror the loading phase trends, where the displacement decreases progressively with unloading.

In fact, in contrast to the loading phase, the z-axis displacement decreases with the reduction in applied load. The displacement curves at various times (up to t = 0.30 s) are still non-linear, but the material returns to its original position.

However, a residual displacement remains after unloading, suggesting that some degree of permanent deformation (plasticity) has occurred, as the material does not fully recover to its original state, as previously observed and discussed for the results of Figure 15 z-axis related to displacement versus the entire simulated time interval.

Like the HYB 0.5% (1:1) formulation, the HYB 0.5% (5:1) formulation does not completely return to its original shape, indicating some level of residual deformation.

However, the residual displacement results were lower compared to the HYB 0.5% (1:1) formulation. This indicates that the HYB 0.5% (5:1) formulation has better recovery characteristics, likely due to its stiffer properties and better deformation resistance.

#### 3.4.3. Von Mises Stress Profiles

**Figure 20:**
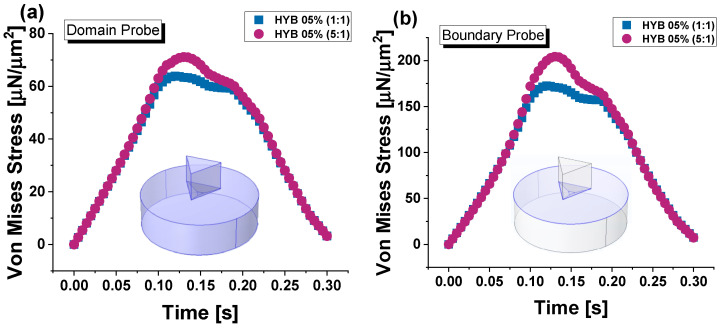
Average Von Mises stress for HYB 0.5% (1:1) and HYB 0.5% (5:1) samples, assessed over the full domain in (**a**) and on the upper surface in (**b**), respectively.

Figure 20 illustrates the progression of average Von Mises stress throughout the entire duration of force application—encompassing the loading, holding, and unloading phases—for both composite materials, HYB 0.5% (1:1) and HYB 0.5% (5:1). Stress values are evaluated across the full material domain in (a) and at the upper surface in (b), as schematically shown in their corresponding insets.

In the domain probe, which may reflect stress deeper within the material, HYB 0.5% (5:1) reaches higher stress values than HYB 0.5% (1:1), peaking around 71 μN/μm^2^. In fact, the overall trend shows that HYB 0.5% (1:1) has a slower rate of stress increase and a lower maximum stress value (64 μN/μm^2^), which aligns with its lower hardness and higher susceptibility to indentation. This difference suggests that the higher MWCNT content in HYB 0.5% (5:1) significantly reinforces the material structure, allowing it to bear a greater load before deformation compared to HYB 0.5% (1:1). For both samples, the highest stress level is observed at t = 0.13 s, which coincides with the maximum indentation depth. In the surface probe evaluation, stress values are notably higher for both materials, with the HYB 0.5% (5:1) sample again reaching greater maximum stress levels, exceeding 200 μN/μm^2^. This heightened surface stress response highlights the impact of MWCNTs in increasing surface hardness and limiting contact depth under load. The HYB 0.5% (1:1) sample, while following a similar trend, reaches lower maximum stress values at the surface (172 μN/μm^2^), indicating less resistance to surface indentation. 

**Figure 21:**
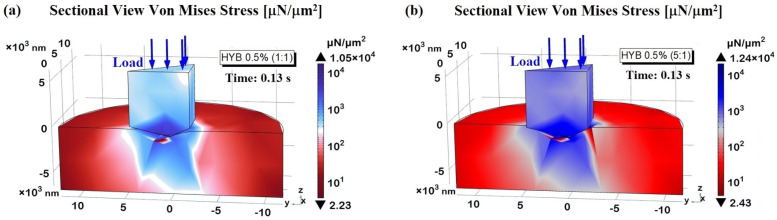
The 3D sectional view of the Von Mises stress measured at t = 0.13 s within the materials, displayed for HYB 0.5% (1:1) in panel (**a**) and HYB 0.5% (5:1) in panel (**b**).

Figure 21 presents 3D visualizations of the two samples, with sections cut to improve the visibility of the Von Mises stress distribution within their respective domains. Panel (a) displays HYB 0.5% (1:1), while panel (b) shows HYB 0.5% (5:1).

These images reveal that the highest stress concentrations are located at the contact interface between the indenter probe and the sample surface, extending outward from the contact point into the surrounding material. This stress gradient demonstrates a progressive decline in intensity with distance from the contact area. Consistent with the findings shown in Figure 21a, the HYB 0.5% (5:1) sample exhibits notably higher stress levels than the HYB 0.5% (1:1) sample, indicating greater resistance to deformation under load due to its composition. This difference highlights the impact of increased MWCNT concentration on stress distribution within the material.

**Figure 22:**
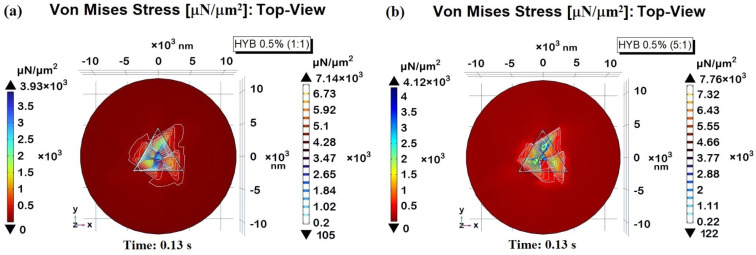
Von Mises stress recorded at the time instant t = 0.13 s for and HYB 0.5% (1:1) in (**a**) and (**b**), respectively.

In conclusion, the Von Mises stress distributions observed on the upper surfaces of the materials are subjected to a more detailed graphical analysis, presented through 3D top views in Figure 22, panels (a) and (b), which correspond to the HYB 0.5% (1:1) and HYB 0.5% (5:1) samples, respectively. These supplementary graphical representations emphasize and quantify the findings, as indicated by the accompanying color bars, illustrating that the highest concentrations of Von Mises stress are localized within the indentation regions. To be precise, since HYB 0.5% (5:1) has higher hardness and reduced modulus, it undergoes less deformation and has a lower contact depth under the same load. This means the material can sustain a higher localized stress (maximum peak of 1.24 × 10^4^ µN/µm^2^) before significant deformation spreads through it. As a result, the stress contours for these formulation results are concentrated in a smaller region near the contact point, creating sharper contour gradients and maintaining higher stress values close to the point of indentation. Conversely, the HYB 0.5% (1:1) material, being softer with higher contact depth, distributes the stress more evenly over a larger area. This is why the contours in the HYB 0.5% (1:1) image appear more spread out, with smoother gradients extending outward, reflecting the material’s tendency to deform more and spread stress over a broader area. For this formulation, the stress values reach a maximum value of 1.05 × 10^4^ µN/µm^2^.

#### 3.4.4. Mechanical Energy Flux and Total Elastic Strain Energy

The z-component of the mechanical energy flux over time reflects how mechanical energy is transferred into and out of the material during loading and unloading. The oscillations stem from the material’s elastic and possibly viscoelastic response to a load function, showing how it absorbs, stores, and releases energy dynamically. 

In our case, where a trapezoidal load function is applied, the graph’s oscillating behavior can be explained as follows:

- Loading Phase (Increasing Slope): At the beginning, as the force gradually increases (the rising slope of the trapezoid), the mechanical energy flux also rises, indicating that energy is being transferred into the material. The flux increases smoothly, reflecting how the material absorbs energy from the applied load.
- Plateau Phase (Constant Load): Once the force reaches its maximum and remains constant (the flat top of the trapezoid), the energy flux stabilizes. The material is subjected to a steady load, and there may still be some minor energy fluctuations, especially due to internal stress redistributions, damping effects, or viscoelastic behavior of the material. The oscillations during this phase could indicate a dynamic response as the material adjusts to the constant force.
- Unloading Phase (Decreasing Slope): As the load decreases during the unloading phase (the descending slope of the trapezoid), the energy flux exhibits negative values, indicating that the material is now returning energy as it elastically recovers. The oscillations in the negative range reflect how the energy is released unevenly as the material tries to regain its original shape, influenced by its elastic and possibly viscoelastic properties.
- Post-Unloading Phase: Once the load is completely removed, the energy flux stabilizes near zero, indicating that no more energy is being transferred to or from the material.

In particular, in a nanoindentation test, the z-component of mechanical energy flux (in W/m^2^) is closely related to how the material deforms and responds to applied loads over time. This response is influenced by the material’s hardness and contact depth, as hardness reflects resistance to deformation, while contact depth measures how deeply the indenter penetrates the material.

**Figure 23:**
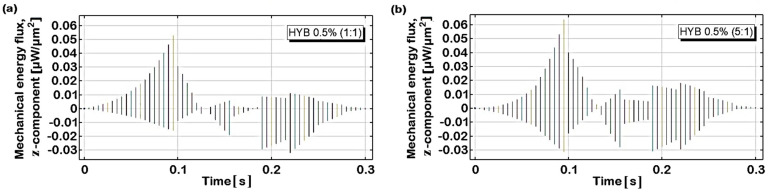
Mechanical energy flux, z-component, transferred during the entire time windows of nanoindentation test for HYB 0.5% (1:1) in (**a**) and HYB 0.5% (5:1) in (**b**).

The graphs of Figure 23 show the mechanical energy flux (z-component, evaluated along the symmetry axis of the system consisting of the sample and the nanoindentation tip) as a function of time for the two samples loaded with 0.5 wt% of filler mix at different compositions: (a) HYB 0.5% (1:1) and (b) HYB 0.5% (5:1). These samples have distinct mechanical properties as observed during nanoindentation, where HYB 0.5% (1:1) is associated with lower hardness and greater contact depth, while HYB 0.5% (5:1) demonstrates higher hardness and reduced contact depth. The mechanical energy flux graphs reflect the mechanical properties identified in the nanoindentation tests. As a consequence, a material with higher hardness, such as the HYB 0.5% (5:1) sample, resists deformation more effectively, leading to less penetration by the indenter and a shallower contact depth. This means that the indenter exerts a larger force over a shorter distance (less depth). The mechanical energy flux (z-component) will exhibit sharper, more intense peaks during loading. Since the material resists indentation, energy builds up quickly as the indenter forces the material to deform elastically. The energy flux will reflect this rapid change, resulting in higher spikes within a shorter time frame. Otherwise, the sample HYB 0.5% (1:1) with lower hardness allows for greater penetration by the indenter, which translates to a larger contact depth. This deeper penetration requires more time and a less intense force to induce deformation. As a result, the mechanical energy flux (z-component) exhibits a broader, less intense profile. The energy is spread out over a longer period of time since the material is easier to penetrate, requiring less force at any given moment to achieve the same indentation. The energy flux curve is more gradual, indicating a slower buildup and release of energy. During the unloading phase, since the material has undergone deeper penetration and may not fully recover elastically, the unloading phase will show a slower decline in energy flux. Some residual energy may remain, indicating partial plastic deformation. As key observations, the energy flux fluctuates symmetrically about the zero point, indicating a dynamic process with alternating periods of positive and negative energy transfer. The peak energy flux occurs near 0.1 s, representing the moment of maximum energy transfer in the system with peak values of about 0.05 µW/µm^2^ for HYB 0.5% (1:1) and more than 0.06 µW/µm^2^ for HYB 0.5% (5:1).

**Figure 24:**
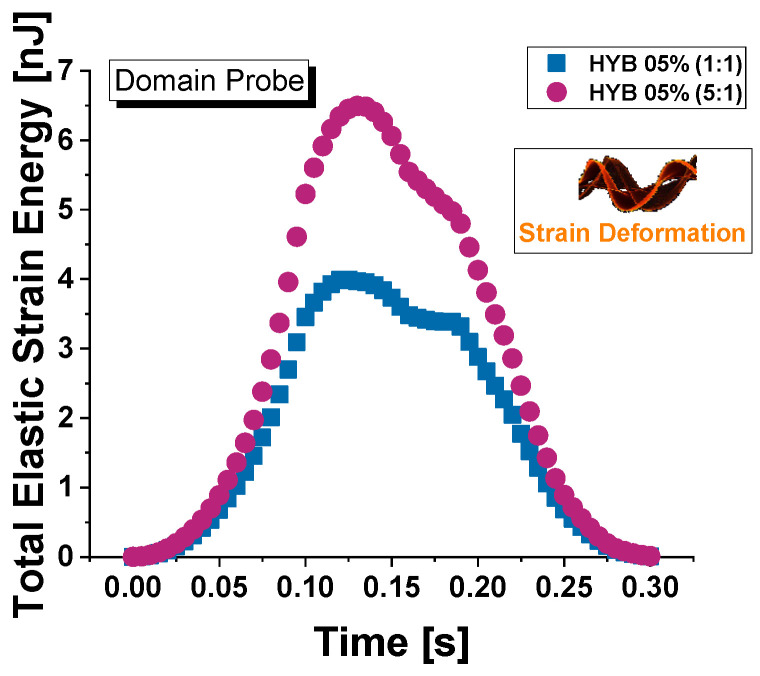
Total elastic strain energy stored in the two formulations, HYB 0.5% (1:1) and HYB 0.5% (5:1), evaluated on the overall volume during the entire time windows of nanoindentation test.

Figure 24 depicts the average total elastic strain energy density (in nJ) as a function of time (in seconds), evaluated on the overall volume domain. The total elastic strain energy density represents the amount of energy stored in a material as a result of its elastic deformation.

With reference to a nanoindentation test, the total elastic strain energy is the energy stored in the material due to elastic deformation as the indenter presses into the surface. This elastic strain energy depends on both the hardness of the material and the contact depth of the indentation.

About the time-dependent behavior, it is possible to note that, initially, during the load phase (0 to ~0.10 s), as time progresses, the elastic strain energy density increases, reaching a maximum value. This increase corresponds to the material being loaded, where elastic energy is stored as the material undergoes deformation. The material absorbs energy as stress builds up due to an applied load, likely a trapezoidal load in the present study. At around 0.13 s, the elastic strain energy density reaches its peak value, approximately 4 nJ and 7 nJ for HYB 0.5% (1:1) and HYB 0.5% (5:1) samples, respectively. This peak marks the point where the material has stored the maximum amount of elastic energy before the load begins to stop or decrease. When the load is released (0.20 s to 0.30 s), this energy is gradually released, resulting in a return to the initial energy level, indicating that the load has been completely removed and the material has either fully or mostly recovered elastically.

According to the nanomechanical properties, the formulation HYB 0.5% (5:1) with a higher hardness value resists plastic deformation more effectively compared to the sample HYB 0.5% (1:1), meaning that a larger proportion of the indentation response is due to elastic deformation. As a result, the elastic strain energy stored in this harder material tends to be higher, and at the same time, the elastic strain energy will reach a higher peak value because more energy is stored elastically in the material before any permanent (plastic) deformation occurs. Differently, softer materials, like HYB 0.5% (1:1), undergo more plastic deformation for a given load. Since elastic recovery is less significant in these materials, the elastic strain energy will be lower compared to harder materials for the same applied load. Consequently, the energy density will have lower peak values because the material accommodates the applied force with more plastic deformation and less elastic recovery.

More considerations and general aspects are provided in the next section, Discussion.

#### 3.4.5. Cauchy-Green Tensor, z-Component

In a nanoindentation test, where small deformations mechanics occur, the Cauchy-Green strain tensor components, specifically the z-component, reflect how the material deforms under the indenter. Specifically, the z-component C_zz_, which measures deformation along the indentation direction (normal to the surface) is analytically expressed by the following relation:

(12) $$C_{zz}={\left(\frac{\partial Z^{\prime }}{\partial Z}\right)}^{2}$$

where Z′ is the deformed coordinate, and Z is the undeformed coordinate.

**Figure 25:**
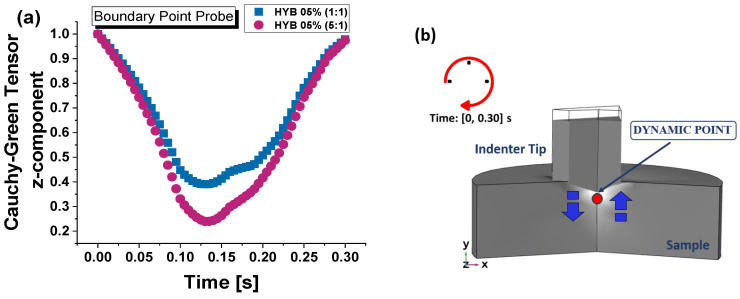
Cauchy-Green tensor, z-component, for HYB 0.5% (1:1) and HYB 0.5% (5:1) samples in (**a**) and schematic representation of the dynamic point at which it is evaluated in (**b**).

The key factors influencing this deformation are hardness and contact depth. Let us break down how these parameters affect the z-component of the Cauchy-Green strain tensor on the basis of numerical results shown in Figure 25 for both formulations HYB 0.5% (1:1) and HYB 0.5% (5:1). The Cauchy-Green tensor, z-component, is evaluated over the entire nanoindentation time interval [0, 0.30] s at the dynamic point (indicated by the red dot in the schematic representation in Figure 25b), which moves within the sample domain at the indentation tip as it penetrates and retracts in response to the applied load.

Harder materials, such as HYB 0.5% (5:1), deform less for a given applied force, leading to smaller displacements. The deformation near the indenter in the z-direction is less for harder materials, which leads to a smaller C_zz_ value (i.e., 0.239). On the contrary, in softer materials like HYB 0.5% (1:1), the indenter penetrates deeper under the same load, producing greater deformation along the z-axis and, hence, a larger C_zz_ (i.e., 0.389).

Greater contact depth means that the material deforms more along the indentation direction. This leads to higher compressive strains, resulting in a larger C_zz_. As the contact depth increases, the strain distribution under the indenter becomes more complex, but overall, the z-component of the Cauchy-Green tensor increases as more material experiences significant deformation. Thus, deeper indentations (as in the case of HYB 0.5% (1:1)) result in a larger contact depth and, hence, in a larger C_zz_ with respect to the HYB 0.5% (5:1) formulation.

**Figure 26:**
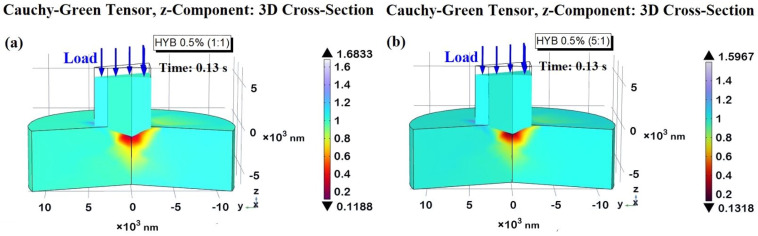
3D cross-sectional views of Cauchy-Green tensor, z-component, for HYB 0.5% (1:1) and HYB 0.5% (5:1) samples in (**a**) and (**b**), respectively.

Finally, to conclude this numerical mechanical investigation, Figure 26 reports a 3D cross-sectional view of the Cauchy-Green tensor (z-component) for two epoxy hybrid samples: HYB 0.5% (1:1) in subplot (a) and HYB 0.5% (5:1) in subplot (b). This visualization allows us to clearly examine the distribution and intensity of the tensor values within each material under applied stress. The color bar and tensor values provide quantitative insight into these material behaviors.

A comparative graphical and numerical analysis based on two formulations highlights that the HYB 0.5% (1:1), with lower hardness and a higher contact depth compared to HYB 0.5% (5:1) in (b), makes this material in (a) more prone to deformation under load, affecting the distribution of the tensor. In HYB 0.5% (5:1), the higher hardness results in less deformation, which is reflected in the tensor distribution and the peak values observed in (b).

The color bar provides a clear indication of the distribution intensity across the samples, with red and orange areas indicating high tensor values and cooler colors representing lower values.

In (a), it is possible to note a more intense red–orange region at the contact area, which extends deeper into the material, consistent with greater deformation. This distribution is spread more widely, illustrating how the softer HYB 0.5% (1:1) absorbs more of the applied stress. In contrast, the tensor distribution in (b) is more contained, with a smaller, less intense red region near the contact area, demonstrating how the harder HYB 0.5% (5:1) confines stress more effectively, resulting in a smaller, more localized high-tensor zone.

Numerically speaking, with reference to HYB 0.5% (1:1), the overall maximum Cauchy-Green tensor value reaches 1.6833, whereas for HYB 0.5% (5:1), it is slightly lower at 1.5967. This numerical difference indicates that HYB 0.5% (1:1) endures a greater deformation response, aligning with its lower hardness. The minimum values are also different, with 0.11884 in (a) and 0.1318 in (b), which corresponds to the higher resilience of the harder HYB 0.5% (5:1) sample.

### 3.5. Morphological Investigation by FESEM and TUNA

**Figure 27:**
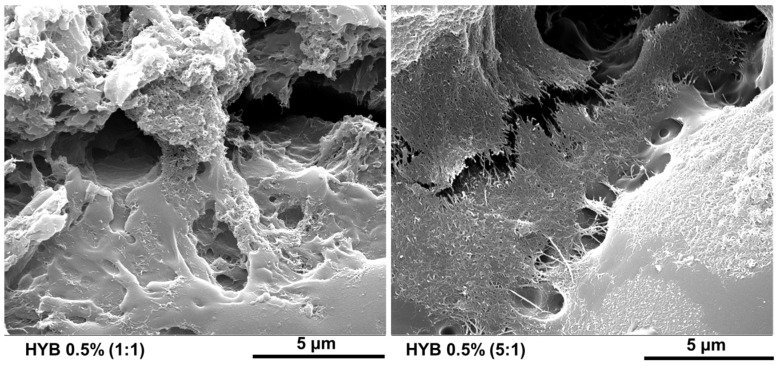
FESEM images of the HYB 0.5% (1:1) and HYB 0.5% (5:1) nanocomposites.

**Figure 28:**
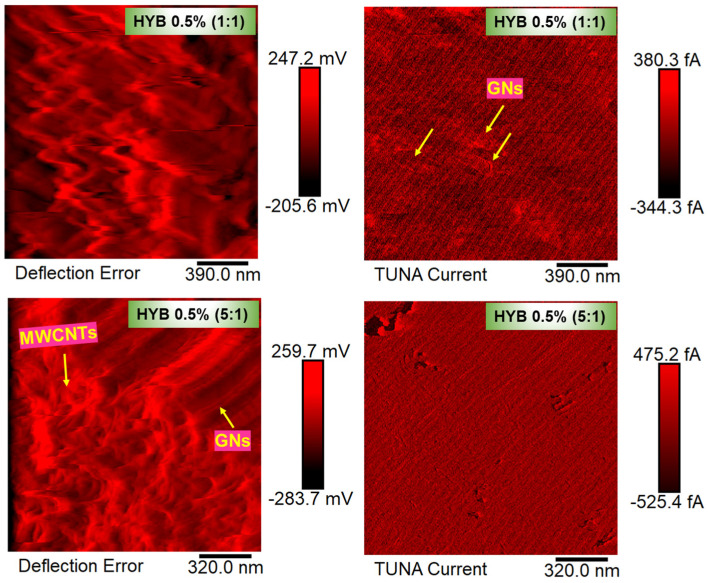
Deflection error and TUNA current images of the HYB 0.5% (1:1) and HYB 0.5% (5:1) nanocomposites.

A morphological analysis of the HYB 0.5% (1:1) and HYB 0.5% (5:1) hybrid nanocomposites was carried out using FESEM and TUNA in order to evaluate the dispersion state of MWCNTs and GNs inside the epoxy matrix, investigate the specific nanofiller–nanofiller and nanofiller–matrix interactions, and comprehend the morphological characteristics of the conductive nanoparticles that contribute to the effective interconnections responsible for the synergistic effect between 1D and 2D fillers through the π–π bond interactions formed between the MWCNTs and the GNs [18]. In this regard, for the HYB 0.5% (1:1) and HYB 0.5% (5:1) nanocomposites, the FESEM images (see Figure 27) and deflection error and TUNA current images (see Figure 28) are shown.

The distribution of the nanofillers within the epoxy matrix and the specific interactions that gave the formulated hybrid samples their exceptional electrical, thermal, and mechanical properties [18,29] were made possible by the oxidizing etching process, which successfully eliminated the amorphous resin. More precisely, the unique configuration of the carbon nanotubes, which appear to be oriented at the interface with the graphene nanoplatelets, facilitates the intense interactions between MWCNTs and GNs. This results in a highly cross-linked three-dimensional network. In Figure 27, we can see that, for both samples, the carbon nanotubes (MWCNTs), which emerge from the epoxy matrix and link with the overlapping graphene nanosheets (GNs), are plainly visible through the dark gray appearance of the resin. The carbon nanotubes are visible, establishing π–π bond interactions on the GNs’ surface. Furthermore, a close inspection of the picture reveals that different graphene nanolayers are connected by the carbon nanotubes, which exhibit high adherence to the GNs and the polymeric matrix, thereby serving as a bridge between the matrix and the graphene layers. In the HYB 0.5% (5:1) sample that was loaded with a higher weight amount of MWCNTs, a dense network of uniformly intertwined carbon nanotubes is clearly observable, which extends across the entire investigated surface. At lower concentrations, when the balance between conductor and insulator is still delicate, this transition takes place. However, the system already has clearly defined conductive pathways at higher MWCNT concentrations; thus, the addition of MWCNTs has less of an effect on the electrical characteristics. It should be noted that only epoxy systems containing graphene nanoparticles exhibit, at concentrations close to the percolation threshold, the particular orientation of the MWCNTs at contact with the GNs. The purpose of TUNA analysis is to map the local electric current values of the conductive nanodomains and to give precise information on the conductive nanofillers present in the host matrix. For each sample, two images representing the same scanned area are shown: TUNA current and deflection error. The deflection error image represents the error signal of the deflection parameter that sets the required voltage (and, therefore, the required cantilever deflection or force) in the feedback circuit.

The deflection error is closely related to the deviation of the vertical deflection from the deflection setpoint that occurs when the tip contacts a particle during the scanning phase of the sample surface.

Under these conditions, the tip bounces slightly upward, causing a slight upward bending of the cantilever with a subsequent increase in vertical deflection.

However, the feedback circuit can effectively act to return the vertical deflection to the nominal value using the gain set by the user and sent to the Z piezo element to move the tip up or down to minimize the error. This type of image helps to discriminate finer details relating to the morphological characteristics of the surface investigated. From Figure 28, we can see that the surface of both samples HYB 0.5% (1:1) and HYB 0.5% (5:1) shows the presence of carbon nanotubes, which is, however, more pronounced for the HYB 0.5% (5:1) sample containing a higher quantity of MWCNTs. The MWCNTs entwine with one another and successfully connect with the graphene nanosheets (GNs), which also form interfacial bonds with the host matrix. The MWCNTs in both TUNA images seem to align themselves at the interface with the discernible edges of the graphene nanosheets (GNs), just like in the FESEM images. TUNA provides information on the greater or lesser local electrical conductivity associated with a range of colors present on the TUNA current image scale bar, which goes from the darkest for less conductive areas to the lightest for more conductive areas. In particular, from TUNA current images of Figure 28, we can see that the HYB 0.5% (1:1) sample shows current values ranging from −344.3 fA to 380.3 fA, which are low compared to the current values between −525.4 fA and 475.2 fA observed for HYB 0.5% (5:1). It is worth noting that the electrical conductivity values at nanoscale level are in agreement with those of the electrical DC conductivity. In fact, the highest value is the one recorded by the HYB 0.5% (5:1) sample, which is equal to 2.94 × 10^2^ S/m, while for the HYB 0.5% (1:1) sample, the value is equal to 9.79 × 10^3^ S/m [18]. According to the TUNA results, electric current values that unquestionably demonstrate the formulated nanocomposites’ inherent electrical conductivity can be detected even if the examined samples are not grounded.

**Figure 29:**
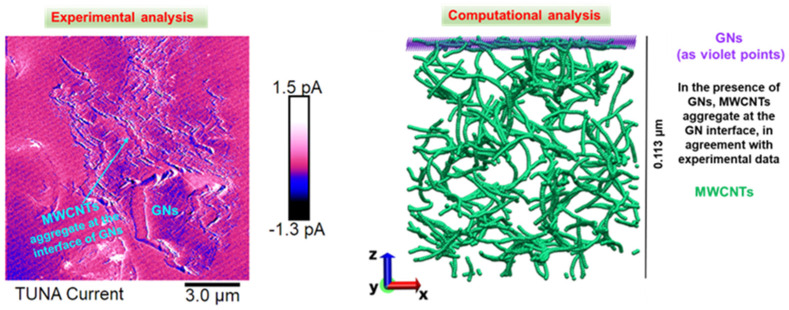
Correlation between experimental and computational results on a micrometric scale for the sample HYB 0.1% (1:5).

It is worth noting that we thoroughly investigated the synergistic effect of the two conductive nanofillers, multi-walled carbon nanotubes (MWCNTs) and graphene nanosheets (GNs), on the electrical properties of nanohybrid systems by a combined computational and experimental approach [18]. The first allows us to understand these materials at the nanoscale, and the second allows us to obtain the electrical mapping of nanometric domains by conducting direct current (DC) measurements and investigation by Tunneling Atomic Force Microscopy (TUNA). The electrical mapping of nanometric domains highlights the presence of a conductive network characterized by high current density. At a low number of nanoparticles, 0.1 wt%, the hybrid nanofiller concentration allows for detecting a very interesting synergy toward an increase in the electrical conductivity of several orders of magnitude and a lowering of the Electrical Percolation Threshold (EPT). Both the computational and experimental results evidence that, owing to the hybrid MWCNT/GNs network formation, the hybrid nanocomposites outperform their single-nanofiller counterparts. In particular, computational analysis showed that MWCNTs aggregated at the GN interface, in agreement with experimental data. In this regard, Figure 29 visually shows the correlation between experimental and computational results on a micrometric scale for the sample HYB 0.1% (1:5). This figure clearly illustrates the interactions and combined effects of these nanofillers on the epoxy matrix. This assembly, not observed in the graphene nanosheet-free composite system, seemed to make the hybrid system more conductive at the MWCNT concentration below the EPT compared to the binary one based on MWCNTs alone. We hypothesized that this behavior is derived from the specific morphology assumed by the MWCNTs at the interface with GNs. 

This synergistic effect of MWCNTs:GNs on the interface performance of hybrid composites paves the way for a research sector where the analysis of this aspect is still lacking, thus allowing us to understand the reason which MWCNTs and GNs are highly effective in enhancing the overall performance of nanocomposites and suitable for a wide range of advanced applications in which carbon-based nanofillers already play a relevant role in impacting the final properties of nanocomposites [44]. Here are some key points regarding the beneficial implications arising from the synergy between MWCNTs and GNs:

- Mechanical Properties: The combination of one-dimensional MWCNTs and two-dimensional graphene nanosheets creates a unique network that improves the mechanical strength and toughness of the nanocomposites. This is due to the high aspect ratio and excellent mechanical properties of both fillers [19,45].
- Electrical Conductivity: The different geometrical shapes of MWCNTs and graphene help form a conductive network within the composite, enhancing its electrical conductivity. This is particularly beneficial for applications requiring efficient electrical pathways [46].
- Thermal Conductivity: Both MWCNTs and graphene have high thermal conductivity. Their synergistic effect can improve the nanocomposites’ heat dissipation properties, making them suitable for thermal management applications [45].
- Fatigue and Fracture Toughness: The hybrid nanofiller system can improve the fatigue life and fracture toughness of the composites. The flexible MWCNTs bridge adjacent graphene sheets, preventing agglomeration and creating a favorable network for load transfer [19].
- EMI Shielding: The combined use of MWCNTs and graphene can also enhance the electromagnetic interference (EMI) shielding effectiveness of the nanocomposites. This is due to the unique conductive network formed by the fillers [46].

Furthermore, the compatibility of epoxy resin with MWCNTs and graphene nanosheets is fundamental to maximizing the performance benefits of these nanofillers. Epoxy resin exhibits excellent compatibility with both multi-walled carbon nanotubes (MWCNTs) and graphene nanosheets (GNs) due to several key factors:

- Functional Groups: The presence of functional groups on CNTs and graphene nanosheets enhances their interaction with the epoxy resin. Functional groups, such as carboxyl, hydroxyl, and amine groups, can form strong covalent bonds with the epoxy matrix, improving interfacial adhesion [47,48].
- Surface Area and Aspect Ratio: Both MWCNTs and GNs have high surface areas and aspect ratios, which provide extensive contact with the epoxy resin. This large interface area facilitates better load transfer and stress distribution within the composite [49,50].
- π–π Interactions: The aromatic rings in the epoxy resin can interact with the π-electron clouds of CNTs and graphene through π–π stacking interactions. These non-covalent interactions further enhance the compatibility and dispersion of the nanomaterials within the epoxy matrix [47,48].
- Dispersion Techniques: Effective dispersion techniques, such as sonication, shear mixing, and the use of surfactants or functionalization, help in uniformly distributing CNTs and graphene within the epoxy resin. This uniform dispersion is crucial for achieving consistent properties throughout the composite [49,50,51,52].
- Mechanical and Thermal Properties: The inherent mechanical strength and thermal conductivity of CNTs and graphene complement the properties of epoxy resin. When combined, they create a composite material with superior mechanical strength, thermal stability, and electrical conductivity [47,50].

These factors, cumulatively, contribute to epoxy resin’s excellent compatibility of epoxy resin with MWCNTs and graphene nanosheets, making them ideal for high-performance nanocomposites.

## 4. Conclusions

Hybrid epoxy systems have become increasingly important in the materials field due to their ability to combine the mechanical strength and chemical resistance of traditional epoxy resins with enhanced properties provided by various fillers. By incorporating additives such as carbon nanotubes (CNTs), graphene nanoparticles (GNs), or other nanomaterials, hybrid epoxy systems can be engineered to achieve unique combinations of mechanical, thermal, electrical, and other physical properties that are difficult to attain with conventional epoxies alone. Nanomechanical testing has become an indispensable tool for exploring the mechanical properties of materials at the nanoscale. This methodology is particularly valuable for composite materials, as it reveals detailed information on mechanical properties like durability, stiffness, strength, and resilience. These insights are instrumental in refining composite formulations and enhancing manufacturing processes.

When applied to composite materials, nanoindentation offers a detailed assessment of the mechanical behavior of the matrix itself, as well as the influence of any functional additives or dispersed phases within it. This is especially valuable for composites, where added phases or agents may significantly alter the mechanical profile of the base material. By conducting nanoindentation tests at multiple locations within a composite, researchers can also gauge the uniformity of properties across different regions, which is essential for understanding material homogeneity. This spatial analysis helps identify any local variations in mechanical properties, ensuring that the composite meets desired performance standards and behaves consistently under various conditions.

The main results are summarized below:

- This study provides an in-depth experimental exploration of hybrid epoxy-based nanocomposites reinforced with multi-walled carbon nanotubes (MWCNTs) and graphene nanosheets (GNs), with a particular numerical focus on two specific formulations: HYB 0.5% (1:1) and HYB 0.5% (5:1), which represent mechanically contrasting systems.
- By combining experimental nanoindentation with advanced numerical simulations, we successfully validated the numerical model through experimental data, ensuring the accuracy of the simulation results.
- The Design of Experiments (DoE) approach was used in the present study to produce two key plots: the Dex Scatter Plot (DSP) and the Main Factor Plot (MFP). These tools visually analyze the impact of various input factors on the performance function (P.F.), particularly the mechanical properties such as reduced modulus, hardness, and contact depth of two composite formulations, HYB 0.1% and HYB 0.5%, with differing weight ratios of multi-walled carbon nanotubes (MWCNTs) to graphene nanosheets (GNs).
- The use of Response Surface Methodology (RSM) helped establish a clear correlation between the filler weight ratio and the aforementioned key mechanical properties. The simulations provided deeper insights into the mechanical behavior of the two extreme samples, particularly their response to loading conditions, revealing the different ways in which MWCNT/GN ratios influence overall material performance.

The HYB 0.5% (1:1) formulation showed relatively lower hardness and greater contact depth, whereas the HYB 0.5% (5:1) demonstrated superior hardness and resistance to penetration. These findings confirm that adjusting the ratio of MWCNTs to GNs significantly alters the mechanical characteristics of the composite, which is crucial for optimizing material design for specific applications. Experimental points align closely with these surfaces, confirming RSM’s predictive accuracy and reliability.

- Ultimately, the study concludes by presenting numerical results obtained through multiphysics simulations conducted with finite element-based software (COMSOL Multiphysics^®^).

In fact, it is widely recognized that simulation studies play a crucial role in understanding the mechanical properties of composite materials, especially at the nanoscale.

Conducting experimental tests on composite materials can be time-consuming, labor-intensive, and expensive.

Simulation studies offer a cost-effective alternative by allowing researchers to explore various design possibilities and hypotheses before manufacturing the composite and experimental testing.

The first step was to ensure the reliability of the numerical results.

They were compared with experimental data for validation.

In particular, the experimental/simulated results regarding the contact depth and hardness of two reference composites, HYB 0.5% (1:1) and HYB 0.5% (5:1), were compared by finding that the values are in perfect agreement. 

These two composites were selected based on experimental characterization as having mechanically less similar properties, and therefore, they were extensively investigated numerically.

- In addition to the mechanical properties experimentally analyzed, further properties were investigated with the validated simulation model.
- In conclusion, simulation studies play a pivotal role in the design of composite materials, offering predictive capabilities, opportunities for optimization, and in-depth insights into the behavior of materials under different conditions.
- By leveraging computational modeling, researchers can accelerate the development process, significantly reduce costs, and tailor composite materials to meet the specific requirements of diverse applications.
- This approach not only enhances the efficiency of materials research but also empowers engineers to design composites with properties precisely suited to their intended uses.

## Figures and Tables

**Table 1:** MWCNTs:GNs mix ratios for hybrid nanocomposites loaded both with 0.1 and 0.5 wt%.

Mix Ratios MWCNTs:GNs	wt% MWCNTs	wt% GNs
1:1	50	50
1:2	33	67
1:5	20	80
2:1	67	33
5:1	80	20

**Table 2:** List of samples and their corresponding physical properties: density, reduced modulus hardness, and contact depth.

Sample	Density [g/cm^3^]	Reduced Modulus [GPa]	Hardness [GPa]	Contact Depth [nm]
HYB 0.1% (1:1)	1.2858	4.32	0.3220	1092.45
HYB 0.1% (1:2)	1.2833	4.28	0.3245	1088.15
HYB 0.1% (2:1)	1.2856	4.29	0.3168	1101.55
HYB 0.1% (1:5)	1.2839	4.25	0.3238	1089.2
HYB 0.1% (5:1)	1.2915	4.37	0.3309	1077.11
HYB 0.5% (1:1)	1.2593	4.45	0.3106	1113.36
HYB 0.5% (1:2)	1.2745	4.28	0.3292	1080.09
HYB 0.5% (2:1)	1.2638	4.24	0.3140	1106.75
HYB 0.5% (1:5)	1.2441	4.41	0.3327	1074.26
HYB 0.5% (5:1)	1.2842	5	0.3716	1014.98

**Table 3:** Coefficients for the pure quadratic response of the nanomechanical properties as determined by RSM.

Property/Sample	β_0_	β_1_	β_2_	β_11_	β_22_	β_12_
Reduced Modulus—HYB 0.1% (MWCNTs:GNs)	+4.4333	−0.0725	−0.0625	+0.0141	+0.0075	0
Reduced Modulus—HYB 0.5% (MWCNTs:GNs)	+5.1683	−0.5575	−0.3300	+0.1158	+0.0533	0
Hardness—HYB 0.1% (MWCNTs:GNs)	+0.3282	−0.0126	+0.0045	+0.0024	−0.0006	0
Hardness—HYB 0.5% (MWCNTs:GNs)	+0.2877	−0.0084	+0.0316	+0.0039	−0.0043	0
Contact Depth—HYB 0.1% (MWCNTs:GNs)	+1081.3510	+22.0350	−7.7875	−4.3116	+1.1625	0
Contact Depth—HYB 0.5% (MWCNTs:GNs)	+1156.9133	+11.3750	−56.7650	−5.9950	+7.8316	0

**Table 4:** Assessment of experimental and simulated results, including relative error percentage (% Change), for FE model validation.

Sample:	Contact Depth Exp. [nm]	Contact Depth Simul. [nm]	% Change	Hardness Exp. [GPa]	Hardness Sim. [GPa]	% Change
HYB 0.5% (1:1)	−1113.36	−1112.7	0.0593	0.3106	0.3290	5.9240
HYB 0.5% (1:1)	−1014.96	−1014.3	0.065	0.3716	0.3960	6.5662

## Data Availability

Data are contained within the article.

## References

[1] Joshi M., Chatterjee U., Rana S., Fangueiro R. (2016). Polymer nanocomposite: An advanced material for aerospace applications. Advanced Composite Materials for Aerospace Engineering Processing, Properties and Applications.

[2] Fan J., Njuguna J. (2016). An introduction to lightweight composite materials and their use in transport structures. Lightweight Composite Structures in Transport.

[3] Hale D.K. (1976). The physical properties of composite materials. J. Mater. Sci..

[4] Bîrcă A., Gherasim O., Grumezescu V., Grumezescu A.M. (2019). Introduction in thermoplastic and thermosetting polymers. Materials for Biomedical Engineering.

[5] Ratna M. (2009). Handbook of Thermoset Resins.

[6] Bhatnagar M.S. (1993). Epoxy resins from 1980 to date. Part 1. Polym. Plast. Technol. Eng..

[7] Li Z., Wang L., Li Y., Feng Y., Feng W. (2019). Carbon-based functional nanomaterials: Preparation, properties and applications. Compos. Sci. Technol..

[8] Geim A.K., Novoselov K.S. (2007). The rise of graphene. Nat. Mater..

[9] Gkourmpis T. (2013). Carbon-based high aspect ratio polymer nanocomposites. Nanoscience and Computational Chemistry.

[10] Midriver G., Jin W. (2021). Carbon nanomaterials: Synthesis, properties and applications in electrochemical sensors and energy conversion systems. Mater. Sci. Eng. B.

[11] Ávila A.F., Carley G., Geraldo V., de Oliveira S. (2013). Multi-phase carbon fiber-MWNT/epoxy composites. Int. J. Compos. Mater..

[12] Dubey R., Dutta D., Sarkara A., Chattopadhyay P. (2021). Functionalized carbon nanotubes: Synthesis, properties and applications in water purification, drug delivery, and material and biomedical sciences. Nanoscale Adv..

[13] Li Y., Yang T., Yu T., Zheng L., Liao K. (2011). Synergistic effect of hybrid carbon nantube–graphene oxide as a nanofiller in enhancing the mechanical properties of PVA composites. J. Mater. Chem..

[14] Zhang C., Ren L., Wang X., Liu T. (2010). Graphene oxide assisted dispersion of pristine multiwalled carbon nanotubes in aqueous media. J. Phys. Chem. C.

[15] Al-Saleh M.H. (2015). Electrical and mechanical properties of graphene/carbon nanotube hybrid nanocomposites. Synth. Met..

[16] Liu H., Gao J., Huang W., Dai K., Zheng G., Liu C., Shen C., Yan X., Guo J., Guo Z. (2016). Electrically conductive strain sensing polyurethane nanocomposites with synergistic carbon nanotubes and graphenebifillers. Nanoscale.

[17] Maiti S., Shrivastava N.K., Suin S., Khatua B.B. (2013). Polystyrene/MWCNT/graphite nanoplate nanocomposites: Efficient electromagnetic interference shielding material through graphite nanoplate–MWCNT–graphite nanoplate networking. ACS Appl. Mater. Interfaces.

[18] Raimondo M., Donati G., Milano G., Guadagno L. (2022). Hybrid composites based on carbon nanotubes and graphene nanosheets outperforming their single-nanofiller counterparts. FlatChem.

[19] Jen Y.-M., Huang J.-C., Zheng K.-Y. (2020). Synergistic Effect of Multi-Walled Carbon Nanotubes and Graphene Nanoplatelets on the Monotonic and Fatigue Properties of Uncracked and Cracked Epoxy Composites. Polymers.

[20] Kim H., Oh E., Hahn H.T., Lee K.-H. (2015). Enhancement of fracture toughness of hierarchical carbon fiber composites via improved adhesion between carbon nanotubes and carbon fibers. Compos. A Appl. Sci. Manuf..

[21] Mittal G., Rhee K.Y., Miškovic´-Stankovic´ V., Hui D. (2017). Reinforcements in multi-scale polymer composites: Processing, properties, and applications. Compos. B Eng..

[22] Yu A., Ramesh P., Sun X., Bekyarova E., Itkis M.E., Haddon R.C. (2008). Enhanced thermal conductivity in a hybrid graphite nanoplatelet–carbon nanotube filler for epoxy composites. Adv. Mater.

[23] Ma P.C., Liu M.Y., Zhang H., Wang S.Q., Wang R., Wang K., Wong Y.K., Tang B.Z., Hong S.H., Paik K.W. (2009). Enhanced electrical conductivity of nanocomposites containing hybrid fillers of carbon nanotubes and carbon black. ACS Appl. Mater. Interfaces.

[24] Prasad K.E., Das B., Maitra U., Ramamurty U., Rao C.N.R. (2009). Extraordinary synergy in the mechanical properties of polymer matrix composites reinforced with 2 nanocarbons. Proc. Natl. Acad. Sci. USA.

[25] Yang S.Y., Lin W.N., Huang Y.L., Tien H.W., Wang J.Y., Ma C.C.M., Li S.M., Wang Y.S. (2011). Synergetic effects of graphene platelets and carbon nanotubes on the mechanical and thermal properties of epoxy composites. Carbon.

[26] Araby S., Saber N., Ma X., Kawashima N., Kang H., Shen H., Zhang L., Xu J., Majewski P., Ma J. (2015). Implication of multi-walled carbon nanotubes on polymer/graphene composites. Mater. Des..

[27] Yue L., Pircheraghi G., Monemian S.A., Manas-Zloczower I. (2014). Epoxy composites with carbon nanotubes and graphene nanoplatelets–Dispersion and synergy effects. Carbon.

[28] Min C., Liu D., Shen C., Zhang Q., Song H., Li S., Shen X., Zhu M., Zhang K. (2018). Unique synergistic effects of graphene oxide and carbon nanotube hybrids on the tribological properties of polyimide nanocomposites. Tribol. Int..

[29] Guadagno L., Naddeo C., Sorrentino A., Raimondo M. (2023). Thermo-mechanical performance of epoxy hybrid system based on carbon nanotubes and graphene nanoparticles. Nanomaterials.

[30] Spinelli G., Guarini R., Ivanov E., Calabrese E., Raimondo M., Longo R., Guadagno L., Vertuccio L. (2024). Nanoindentation Response of Structural Self-Healing Epoxy Resin: A Hybrid Experimental–Simulation Approach. Polymers.

[31] Čech J., Haušild P., Kovářík O., Materna A. (2016). Examination of Berkovich Indenter Tip Bluntness. Mater. Des..

[32] Khelifa M., Fierro V., Celzard A. (2014). Finite Element Simulation of Nanoindentation Tests Using a Macroscopic Computational Model. J. Mech. Sci. Technol..

[33] Troyon M., Huang L. (2005). Correction Factor for Contact Area in Nanoindentation Measurements. J. Mater. Res..

[34] Lee J.H., Lee H., Kim D.H. (2008). A Numerical Approach to Evaluation of Elastic Modulus Using Conical Indenter with Finite Tip Radius. J. Mater. Res..

[35] Kim J.H., Kim D.Y., Lee J., Kwon S.W., Kim J., Kang S.K., Hong S., Kim Y.C. (2024). Elastic Modulus Prediction from Indentation Using Machine Learning: Considering Tip Geometric Imperfection. Met. Mater. Int..

[36] Doerner M.F., Nix W.D. (1986). A Method for Interpreting the Data from Depth-Sensing Indentation Instruments. J. Mater. Res..

[37] Kuehl R.O. (1999). Design of Experiment: Statistical Principles of Research Design and Analysis.

[38] Khuri A.I., Ghosh S., Rao C.R. (1996). Multiresponse surface methodology. Design and Analysis of Experiments. Handbook of Statistics.

[39] Draper N.R., Lin D.K.J., Ghosh S., Rao C.R. (1996). Response surface designs. Design and Analysis of Experiments. Handbook of Statistics.

[40] Raissi S., Farsani R.E. (2009). Statistical process optimization through multi-response surface methodology. Int. J. Comput. Math..

[41] Spinelli G., Guarini R., Guadagno L., Vertuccio L., Romano V. (2024). Thermo-Mechanical and Thermo-Electric Properties of a Carbon-Based Epoxy Resin: An Experimental, Statistical, and Numerical Investigation. Materials.

[42] Oliver W.C., Pharr G.M. (2004). Measurement of Hardness and Elastic Modulus by Instrumented Indentation: Advances in Understanding and Refinements to Methodology. J. Mater. Res..

[43] Sneddon I.N. (1965). The Relation between Load and Penetration in the Axisymmetric Boussinesq Problem for a Punch of Arbitrary Profile. Int. J. Eng. Sci..

[44] Donchak V., Stetsyshyn Y., Bratychak M., Broza G., Harhay K., Stepina N., Kostenco M., Voronov S. (2021). Nanoarchitectonics at surfaces using multifunctional initiators of surface-initiated radical polymerization for fabrication of the nanocomposites. Appl. Surf. Sci. Adv..

[45] Jiang X., Song T., Shao Z., Liu W., Zhu D., Zhu M. (2017). Synergetic Effect of Graphene and MWCNTs on Microstructure and Mechanical Properties of Cu/Ti_3_SiC_2_/C Nanocomposites. Nanoscale Res. Lett..

[46] Sharma S.K., Gupta V., Tandon R.P., Sachdev V.K. (2016). Synergic effect of graphene and MWCNT fillers on electromagnetic shielding properties of graphene–MWCNT/ABS nanocomposites. RSC Adv..

[47] Roy S., Petrova R., Mitra S. (2018). Effect of carbon nanotube (CNT) functionalization in epoxy-CNT composites. Nanotechnol. Rev..

[48] Yao D., Feng C., Pan X., Fan R., Zhan Z., Chen J., Zheng J., Ming P., Sun H., Pei W., Dong Y., Yu H., You S. (2024). Compatibilization of Carbon/Polymer Composites: Molecular Dynamics Simulation. Proceedings of the 10th Hydrogen Technology Convention (WHTC).

[49] Wazalwar R., Sahu M., Raichur A.M. (2021). Mechanical properties of aerospace epoxy composites reinforced with 2D nano-fillers: Current status and road to industrialization. Nanoscale Adv..

[50] Chai S., Liu J., Hou D., Wang P. (2023). Molecular insights into the interfacial adhesion mechanism between carbon nanotubes and epoxy resin. RSC Adv..

[51] Guadagno L., Raimondo M., Vertuccio L., Mauro M., Guerra G., Lafdi K., De Vivo B., Lamberti P., Spinelli G., Tucci V. (2015). Optimization of graphene-based materials outperforming host epoxy matrices. RSC Adv..

[52] Guadagno L., Raimondo M., Vertuccio L., Naddeo C., Barra G., Longo P., Lamberti P., Spinelli G., Nobile M.R. (2018). Morphological, rheological and electrical properties of composites filled with carbon nanotubes functionalized with 1-pyrenebutyric acid. Compos. Part B Eng..

